# Microscopic observations of RGB circularly polarized luminescence from solid microspheres with liquid crystalline molecular order

**DOI:** 10.1080/14686996.2025.2509486

**Published:** 2025-05-28

**Authors:** Kun Li, Chunya Fu, Hiroshi Yamagishi, Sota Nakayama, Wey Yih Heah, Yixiang Cheng, Reiko Oda, Wijak Yospanya, Yohei Yamamoto

**Affiliations:** a Department of Materials Science, Institute of Pure and Applied Sciences, and Tsukuba Research Center for Energy Materials Science (TREMS), University of Tsukuba, Tsukuba, Japan; b State Key Laboratory of Analytical Chemistry for Life Science, School of Chemistry and Chemical Engineering, Nanjing University, Nanjing, China; c Univ. Bordeaux, CNRS, Bordeaux INP, CBMN, UMR 5248, Pessac, France; d Advanced Institute for Materials Research (AIMR), Tohoku University, Sendai, Japan

**Keywords:** Liquid crystal, circularly polarized luminescence, microspheres

## Abstract

Micro-particles with an internal helical liquid crystalline (LC) molecular order serve as efficient and highly compact circularly polarized luminescence (CPL) emitters. However, the coupling between CPL emission and the interior LC molecular order remains poorly understood at the single particle level. Here, we synthesized microspheres from an LC monomer RM23 together with a fluorescent dye and a chiral additive (R/S-BPy) and investigated their CPL properties. Polarized optical microscopy and angle-dependent CPL observations at a single-particle level revealed randomly distributed one-handed helical domains in each sphere, leading to CPL emission with an average dissymmetry factor value |*g*_lum_| of 0.05 regardless the observation angle. The color of the CPL emission is tunable in the range of 450–700 nm by varying the fluorescent dyes doped in the spheres.

## IMPACT STATEMENT

We have developed robust solid microspheres with liquid crystalline molecular order showing angle-independent circular polarized luminescence with tunable color.

## GRAPHICAL ABSTRACT

**Figure:**
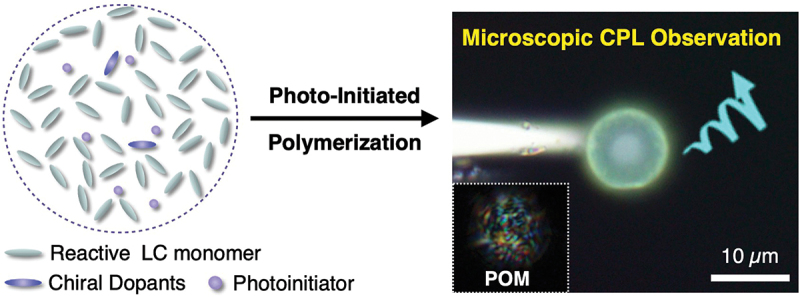

## 1. Introduction

Circularly polarized luminescence (CPL) has drawn increasing attention for its potential applications in 3D displays, telecommunications, encryption, and other related fields [1–4]. Authentic CPL-active materials include chiral organic molecules, lanthanide metal complexes, and polymers [5–7]. Liquid crystalline (LC) materials are another class of CPL-active martials, forming helical molecular organization [8–11]. The handedness of these molecular helices can be biased by adding small portion of chiral molecules, resulting in one-handed helices that scatter preferentially either right- or left-handed CPL, and thereby serve as an efficient CPL material with high luminescence dissymmetry factors (*g*_lum_) [12–15]. Solid spherical particles with LC molecular order are particularly attractive candidates for the CPL micro-emitter due to their unique advantages [16–18]. Unlike pure LC droplets, solid particles are robust, easy to handle and readily integrated in optoelectronic devices. The spherical surface does not significantly affect the angular isotropies of the radiation intensities and *g*_lum_ values, which is in clear contrast with the conventional flat LC films that perturbate the intensities and polarization through reflection and refraction. Moreover, the spherical morphology of these particles sometimes competes with helical molecular architecture and spontaneously forms topological defects that emit extraordinary radiation toward a specific direction. Thus, the angle dependency of the CPL radiation of spherical solid particles is of fundamental interest. For instance, our group reported polymeric microspheres featuring a twisted bipolar molecular arrangement that radiates angularly anisotropic CPL [19,20]. Zhao’s group realized high-performance circularly polarized lasers from single chiral microcrystals [21–23]. Nevertheless, studies on CPL radiation at single-particle level remain far less explored compared to macroscopic counterparts.

**Figure 1.**
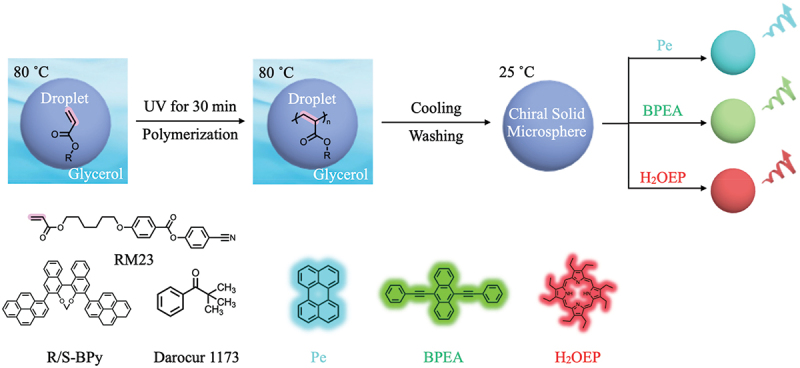
Molecular structure of RM23, R/S-BPy, and Darocur 1173, which can form chiral solid microspheres after exposing UV light. Pe, BPEA, and H_2_OEP were selected as achiral fluorescent molecules, and RGB CPL can be observed by doping them into chiral solid microspheres.

In this study, solid LC microspheres were successfully synthesized by photo-polymerization method (Figure 1). 4-Cyanophenyl-4-((6-(acryloyloxy)hexyl)oxy)benzoate (RM23), (R)/(S)-3,3’-di(1-pyrene)-[1,1’−binaphthalene]-[1,2,1,2-def] [1,3] dioxepine (R/S-BPy), and Darocur 1173 are chosen as reactive LC, chiral dopants, and photo-initiator, respectively. The glycerol dispersion of the particles displayed CPL signals with dissymmetry factor |*g*_lum_| = 0.04. The color of the CPL emission is tunable within the 450 to 700 nm range by incorporating various fluorescent dyes such as perylene (Pe), 9,10-Bis(phenylethynyl)anthracene (BPEA), and 2,3,7,8,12,13,17,18-Octaethylporphyrin (H_2_OEP) (Figure 1). We investigated the angle-dependent CPL radiation from a single particle and revealed angularly isotropic CPL with a |*g*_lum_| value of 0.05. The chiral solid microspheres with LC molecular order radiating isotropic CPL represent promising candidates for the miniaturization of CPL-based optical devices.

## 2. Results and discussion

### 2.1. Preparation and characterization of solid microspheres with LC mesogens

The chiral solid microspheres consisting of LC mesogens were synthesized according to a previous literature [24], and the synthetic procedures are schematically shown in Figure 1. In this work, RM23 and Darocur 1173 were used as the reactive LC mesogen and photo-initiator, respectively. To develop the one-handed helical structure, 3% R- or S-BPy was doped into the microspheres. Hereafter, the resultant microspheres doped with R- and S-BPy are denoted as **LCM**^**R-BPy**^ and **LCM**^**S-BPy**^, respectively.

**Figure 2.**
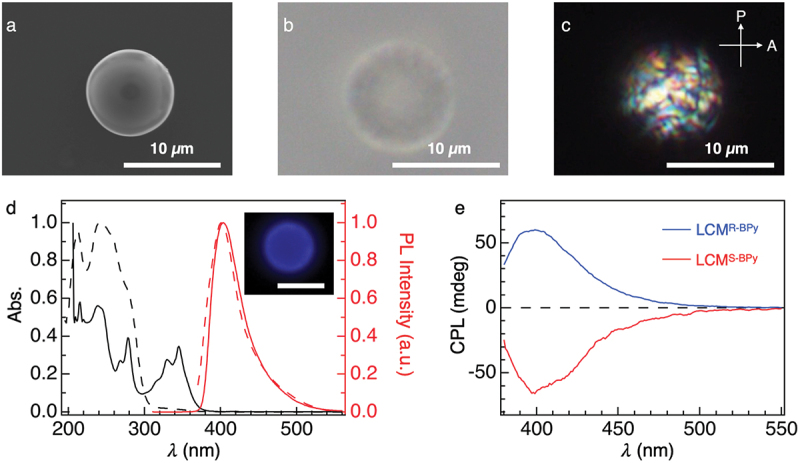
(a–c) SEM micrograph (a), optical micrograph (b), and POM micrograph (c) of **LCM^R-BPy^**. (d) Electronic absorption (black line) and PL (red line) spectra of of R-BPy in CH_2_Cl_2_ (solid line, λ_ex_ = 285 nm) and in **LCM^R-BPy^** dispersion in glycerol (dot line, λ_ex_ = 350 nm). Inset shows the fluorescence microscopy image of **LCM^R-BPy^** (scale bar: 10 µm). (e) CPL spectra (λ_ex_ = 350 nm) of **LCM^R-BPy^** (blue) and **LCM^S-BPy^** (red) dispersed in glycerol.

Optical and scanning electron microscopic (OM and SEM) images of the air-dried precipitates reveal discrete microspheres with a smooth surface (**LCM**^**R-BPy**^, Figure 2a,b) with an average diameter (*d*_av_) of 12.3 µm (Figure S1). Polarized optical microscopic (POM) images of **LCM**^**R-BPy**^ display multiple LC domains (Figure 2c). The brightness and contrast of the **LCM**^**R-BPy**^ under the cross-polarized condition are independent on the in-plane rotation of the sample, indicating the circular birefringence of the microsphere (Figure S2).

The electronic absorption spectrum of **LCM**^**R-BPy**^ in glycerol dispersion shows absorption bands centered at 212, 240, and 340 nm (Figure 2d). The photoluminescence (PL) spectrum showed an emission band at 400 nm, which is consistent with its solution-state emission [25]. The large gap between the absorption and emission bands was attributed to the attenuation of the band at 340 nm from R-BPy due to the intense absorption bands at 240 nm from the liquid crystal polymer. As shown in Figure 2e, the glycerol suspension of **LCM**^**R-BPy**^ shows a remarkable positive CPL band in the 390–420 nm range with a maximum *g*_lum_ value of +0.038 (Figure. S3, λ_ex_ = 350 nm). In contrast, **LCM**^**S-BPy**^ displays a mirror image CPL profile with a minimum *g*_lum_ value of −0.022 confirming the enantiomeric nature of the microspheres [26]. The profiles of *g*_lum_ did not change significantly when changing the concentration of the chiral dopant (Figure S4), which is plausibly because the randomly oriented helical domains scatter the light with less wavelength dependency.

### 2.2. CPL radiation from individual LCM^R/S-BPy^

**Figure 3.**
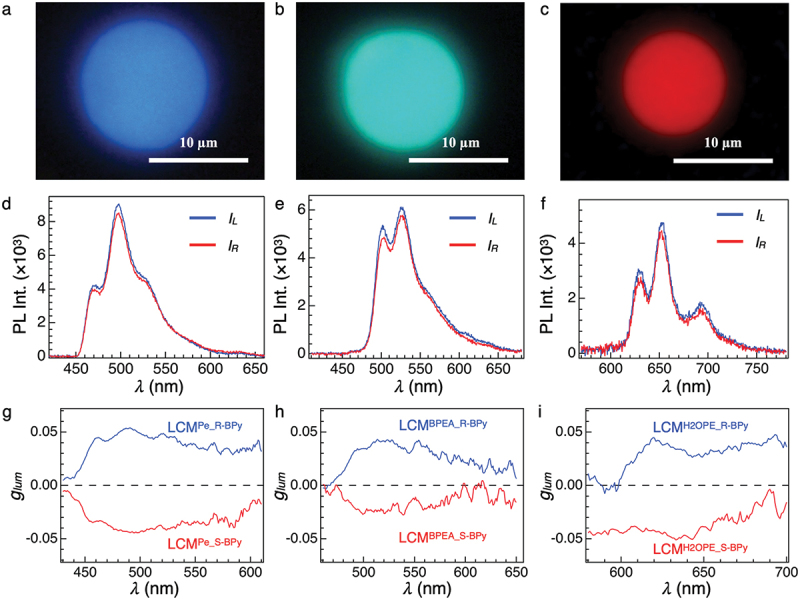
(a – c) fluorescence microscopic images of **LCM**^**Pe_R-BPy**^ (a), **LCM**^**BPEA_R-BPy**^ (b), and **LCM**^**H2OEP_R-BPy**^ (c). (d – f) μ-CPL spectra of a single **LCM^Pe_R-BPy^** (d), **LCM**^**BPEA_R-BPy**^ (e), and **LCM**^**H2OEP_R-BPy**^ (f) with the direction of the polarizer at + 45° (blue, *I*_L_) and − 45° (red, *I*_R_). a single microsphere was excited by a depolarized CW laser (λ_ex_ = 405 nm). (g – i) *g*_lum_ spectra of **LCM^Pe_R/S-BPy^** (g) (λ_ex_ = 390 nm), **LCM^BPEA_R/S-BPy^** (h) (λ_ex_ = 425 nm), and **LCM^H2OEP_R/S-BPy^** (i) (λ_ex_ = 405 nm) suspended in glycerol.

Red-, green-, and blue-color CPL radiation from **LCM**^**R/S-BPy**^ were achieved by introducing Pe, BPEA, and H_2_OEP, into **LCM**^**R/S-BPy**^, which are hereafter termed **LCM**^**Pe_R-BPy**^, **LCM^BPEA_R-BPy^,** and **LCM**^**H2OEP_R-BPy**^, respectively. The fluorescence microscopic images of these microspheres show light-blue, green, and red fluorescence, respectively, indicating Pe, BPEA, and H_2_OEP are successfully doped into **LCM**^**R-BPy**^ (Figure 3a–c). Optical micrographs reveal that the microspherical morphology of **LCM**^**R-BPy**^ is preserved even after doped by the fluorescent dyes (Figure S5), meanwhile their surface is slightly colored by the dyes. POM images confirm that the LC domains as well as circular birefringence are also preserved (Figures S6-S8).

To investigate the CPL properties at the single-particle level, we performed microscopic CPL (μ-CPL) measurements using a homemade spectroscopy setup (Figure S9). A single microsphere was excited by a depolarized CW laser (λ_ex_ = 405 nm). Figures 3d-f and S10 display left-handed (*I*_L_, blue) and right-handed CPL (*I*_R_, red) profiles. **LCM**^**PE_R-BPy**^ displays a slightly stronger left-handed CPL than the right-handed CPL with the *g*_lum_ value of +0.05 at 495 nm. Similarly, **LCM**^**BPEA_R-BPy**^ and **LCM**^**H2OEP_R-BPy**^ display stronger left-handed CPL with a *g*_lum_ value of +0.05 at 525 and 625 nm, respectively. Conversely, **LCM**^**Pe_S-BPy**^, **LCM^BPEA_S-BPy^,** and **LCM**^**H2OEP_S-BPy**^ emit right-handed CPL with *g*_lum_ value of −0.05 at 495, 525, and 625 nm, respectively. To ensure reproducibility, µ-CPL measurements of more than 30 particles were conducted, confirming that **LCM**^**dyes_R-BPy**^ and **LCM**^**dyes_S-BPy**^ display mirror-image *g*_lum_ spectra as shown in Table S1 and Figure S11. It is worth noting that the |*g*_lum_| value is not dependent on the diameter of microspheres (Figure S12). The standard deviations of **LCM**^**Pe_BPy**^, **LCM^BPEA_BPy^,** and **LCM**^**H2OEP_BPy**^ are 0.0124, 0.0171, and 0.0195, respectively (Figure S13). While the signs of the *g*_lum_ values of the glycerol suspension coincide with those obtained from µ-CPL (Figure 3g-i), supporting the validity of the µ-CPL spectra, the absolute |*g*_lum_| values detected with the μ-CPL system are higher than those observed in the glycerol suspension. This indicates that the suspension contains microparticles with less ordered helical molecular arrangement.

### 2.3. Isotropic or anisotropic microspheres

**Figure 4.**
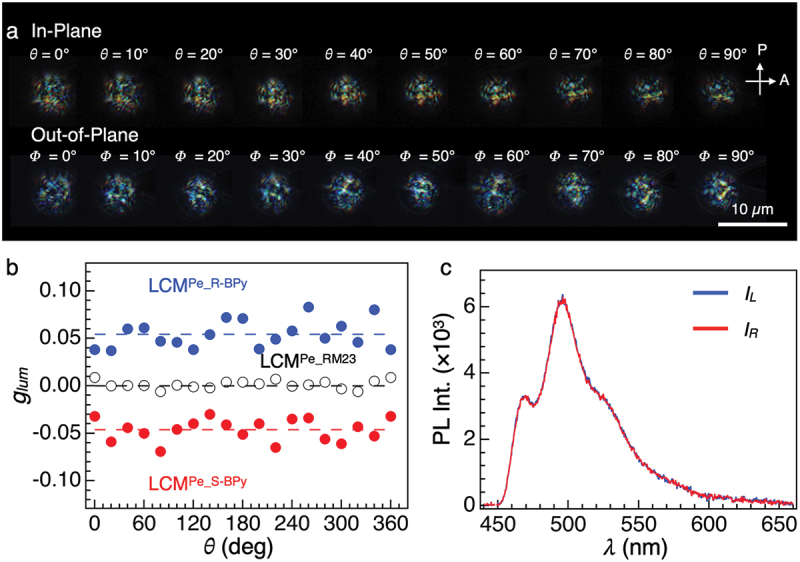
(a) Micrographs of angle-dependent POM textures of a single **LCM**^**Pe_R-BPy**^ operated at in-plane rotation (top) and out-of-plane rotations (bottom). (b) Plots of *g*_lum_ value at 495 nm as a function of θ for **LCM^Pe_R-BPy^** (blue solid circles), **LCM^Pe_RM23^** (black hollow circles), and **LCM**^**Pe_S-BPy**^ (red solid circules), respectively. (c) μ-CPL spectra of a single **LCM**^**Pe_RM23**^ with the direction of the polarizer at + 45° (blue, *I*_L_) and − 45° (red, *I*_*R*_). A single microsphere was excited by a depolarized CW laser (λ_ex_ = 405 nm).

To investigate the helical molecular orientation within the microspheres, we picked up a single microsphere with a sharp tungsten needle and observed its angel-dependent POM textures upon rotating along in- and out-of-plane directions (Figures 4a, S14–S16). The multidomain texture of **LCM**^**Pe_R-BPy**^ remained virtually unchanged upon in-plane and out-of-plane rotation. Similarly, **LCM**^**Pe_S-BPy**^ displays analogous textures for all observed angles (Figures S17 and S18).

We conducted angle-dependent µ-CPL measurements following our previous reports [19,20]. As shown in Figure 4b, *g*_lum_ value of **LCM**^**Pe_R-BPy**^ ranged from +0.04 to +0.08 at 495 nm upon in-plane rotation with the average *g*_lum_ value of +0.057 with a standard deviation of 0.012. Likewise, **LCM**^**Pe_S-BP**^ exhibited a mirror image *g*_lum_ profile with an average *g*_lum_ value of −0.047 the standard deviation of 0.012, respectively. Additionally, the angular-dependent µ-CPL profiles upon out-of-plane rotation were measured (Figure S19). The average *g*_lum_ values of +0.054 and −0.046 were obtained for **LCM**^**Pe_R-BPy**^ and **LCM**^**Pe_S-BPy**^ with the standard deviations of 0.0142 and 0.0115, respectively. Altogether, we confirmed that the |*g*_lum_| values of individual microspheres are angularly isotropic. In theory, the |*g*_lum_| value is highly dependent on the angle between the helical axis and the excitation light [19,27]. The angular isotropic properties obtained both from POM images and from µ-CPL measurements for **LCM**^**Pe_R-BPy**^ and **LCM**^**Pe_S-BPy**^ indicate that the helices are not aligned uniaxially but oriented randomly in the microspheres.

### 2.4. Formation process of the isotropic chiral microsphere

To investigate the influence of *R*- and S-BPy on the helical structure, we synthesized microspheres without chiral dopants, R- or S-BPy, incorporating only Pe, denoted as **LCM**^**Pe_RM23**^ (Figure S20a). These microspheres exhibited light-blue fluorescence and clear birefringence (Figure S20b and c). The birefringence texture of **LCM**^**Pe_RM23**^ were completely different from that of **LCM**^**R-BPy**^, indicating that the chiral dopants influence the molecular arrangement [15,28]. The CPL spectrum of **LCM**^**Pe_RM23**^ in glycerol dispersion does not display any detectable signal (Figure S21). Also, the single-particle of **LCM**^**Pe_RM23**^ displays no CPL activity at 495 nm, showing nearly identical profiles for the polarizer oriented at +45° or −45° (Figure 4c). Angular-dependent µ-CPL measurements does not detect the distinguishable |*g*_lum_| value (Figure 4b). Altogether, we revealed that the chiral source plays a key role in the formation of the helical molecular orders and for generating CPL activity.

**Figure 5.**
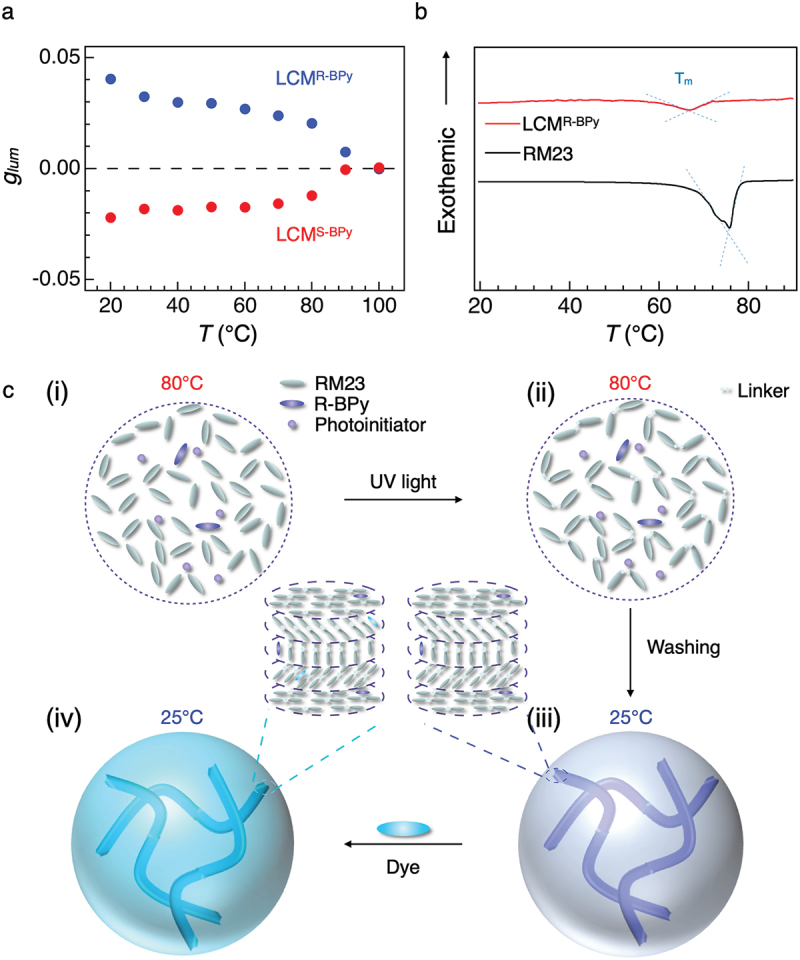
(a) Temperature-dependent *g*_lum_ for **LCM^R-BPy^** (blue) and **LCM^S-BPy^** (red). (b) DSC traces obtained for RM23 (black) and **LCM^R-BPy^** (red). (c) The schematic illustration on the formation process of solid microspheres with LC molecular order. (i) Liquid droplets of monomer in glycerol, (ii) liquid droplets of polymerized monomers in glycerol, (iii) solid microspheres with a helical molecular order, and (iv) solid microspheres doped with a dye.

To gain insight into the formation process of the helical molecular orders in **LCM**^**R-BPy**^, we performed variable-temperature CPL (VT-CPL) spectroscopy, optical microscopy, and POM imaging. VT-CPL spectra of **LCM**^**R-BPy**^ were recorded as the temperature was increased from 20°C to 100°C with 10°C intervals (Figures 5a and S22). The *g*_lum_ values decreased slightly at 30°C and remained constant between 40°C and 70°C and finally dropped to 0 at 90°C (Figure 5a). The *g*_lum_ values of **LCM**^**R-BPy**^ and **LCM**^**S-BPy**^ with different luminophore also vanished at 90°C (Figure S23), indicating the destruction of the helical order in the microspheres. Consistently, variable-temperature optical micrographs POM images also presented clear temperature-dependent changes (Figure S24). The surface darkened at 40°C and the size of the microspheres increased with temperature. The birefringence textures remained up to 70°C and finally disappeared at 90°C.

Differential scanning calorimetry (DSC) profiles revealed that the melting point (*T*_m_) of RM23 is 76°C (Figure 5b), while *T*_m_ of **LCM**^**R-BPy**^ is slightly lower (67°C). In the variable temperature X-ray diffraction (VT-XRD) patterns, diffraction peaks at around 2θ = 23° for **LCM**^**R-BPy**^ were more intense and wider compared to that of **LCM**^**RM23**^ (Figure S25). A well-defined diffraction corresponding to the *d*-spacing of 3.86 Å is ascribed to π–π* stacking [29,30], confirming well-ordered molecular alignment of **LCM**^**R-BPy**^. The intensity at around 2θ = 23° was dependent on temperature and the peak became more intense between 45°C and 65°C. Such evolution of the diffraction intensity for **LCM**^**R-BPy**^ may originate from the better molecular alignment with increasing temperature, due to the molecular motion of LC mesogens.

Based on the above analysis, the formation process of the isotropic chiral microsphere is proposed as shown in Figure 5c. In glycerol, the reactive LC, chiral source, and photo-initiator are mixed together and form microspheres due to surface tension [26, 31,32]. Photopolymerization proceeds under UV light at 80°C to interconnect the randomly dispersed molecules. Subsequent slow cooling gradually solidifies the molecular organization and locks the LC domains in the spheres. Simultaneously, randomly oriented multi-domain molecular helices develop with the help of the chiral source, ultimately defining their CPL properties.

## 3. Conclusions

The solid microspheres with helical molecular order containing RM23 and R- or S-BPy as reactive LC and chiral source were synthesized. The random orientation of the helical axis results in angularly isotropic CPL radiation. Doping of fluorescent molecules with blue to red fluorescent colors is successfully achieved, generating RGB CPL with |*g*_lum_| value of 0.04. This work demonstrates the CPL properties of individual microspheres with LC molecular order and achieves RGB CPL emitting on micrometer scale, providing a promising platform for miniaturized CPL emitter in various applications.

## 4. Experimental section

### 4.1. Preparation of chiral solid microspheres

The chiral solid microspheres were fabricated according to reported procedures [16]. First, the mixture of RM23 (100 mg), chiral dopants (3 mg), and photoinitiator (Darocur 1173, 12 mg) was heated to 80°C to reach a liquid phase and agitated for 1 min to mix evenly. A few drops of the LC monomer mixture were added in a 20 mL vial with 5 mL of heated glycerol (80°C). The mixture was then exposed to UV light for 30 min at 80°C. Finally, the chiral solid microspheres were obtained by cooling the mixture to 25°C.

### 4.2. Preparation of chiral microspheres with achiral fluorescent molecules

The chiral solid microspheres with blue, green, and red fluorescence were prepared as the above process. For example, the mixture of RM23 (100 mg), chiral dopants (3 mg), Pe (1 mg) and Darocur 1173 (12 mg) was heated to 80°C to reach to liquid phase and agitated for 1 min to mix evenly. A few drops of the LC monomer mixture were then added in 20 ml vial with 5 mL of hot 80°C glycerol. The mixture was exposed to UV light (Hamamatsu Model: C11924–121) for 30 min at 80°C. Finally, the chiral solid microspheres with blue fluorescence were obtained by cooling the mixture to 25°C.

### 4.3. Preparation of individual microspheres

The microspheres were washed by Milli-Q water for l0 times to remove glycerol. Then, the microspheres were cast onto a glass plate. Their size and morphology were observed with optical microscope. In the meantime, the microsphere was moved to another glass plate by using a sharp tungsten needle that was controlled numerically by a micromanipulator (MicroSupport Model, Quick Pro) for CPL measurements.

## Supplementary Material

Supplemental Material
