# Supplementary material for: Microscopic observations of RGB circularly polarized luminescence from solid microspheres with liquid crystalline molecular order
Source: Sci Technol Adv Mater. 2025 May 28;26(1):2509486. doi: 10.1080/14686996.2025.2509486 (PMC12160324; doi:10.1080/14686996.2025.2509486)
Supplement: Supplemental Material [file TSTA_A_2509486_SM2043.docx]

Supporting Information

**Microscopic observations of RGB circularly polarized luminescence from solid microspheres with liquid crystalline molecular order**

Kun Li, Chunya Fu, Hiroshi Yamagishi*, Sota Nakayama, Wey Yih Heah, Yixiang Cheng*, Reiko Oda, Wijak Yospanya, Yohei Yamamoto*

Correspondence to: yamagishi.hiroshi.ff@u.tsukuba.ac.jp (H.Y.), yxcheng@nju.edu.cn (Y. C.), yamamoto@ims.tsukuba.ac.jp (Y.Y.)

Table of Contents

Materials and Methods

Supplementary Figures S1 to S25

Supplementary Tables S1

Supplementary References

# Materials and Methods

All chemicals and reagents were purchased from Sigma-Aldrich, TCI, BLD Pharmatech Ltd., and Fujifilm Wako Chemicals and Biosolve B.V. Unless otherwise noted, all reagents and solvents were used as received. Electronic absorption and fluorescence spectra were measured on a UVvisible spectrometer (JASCO model UV-570) and a fluorescent spectrometer (JASCO model FP6200), respectively. Scanning electron microscopy (SEM) was performed on a Hitachi Model S3700N SEM operating at 15 kV. Optical microscopy (OM), fluorescence microscopy (FM) and polarized optical microscopy (POM) observations were carried out using an Olympus model BX53 upright microscope. Circularly polarized luminescence (CPL) spectra of suspensions of microspheres were obtained using JASCO CPL-300 spectrophotometer. The *g*_lum_ spectra were transferred from CPL spectra using the SpectraManager software of JASCO. Variable temperature CPL (VT-CPL) spectra directly used variable temperature accessories, and the CPL spectra were measured in situ with the change of temperature. CPL measurements of a single microsphere were carried out with home-built μ-PL setup according to our previous report.

# Synthesis of R/S-BPy

**R- and S-BPy** were synthesized according to our earlier report^[S1]^ and fully characterized by ^1^H

NMR.


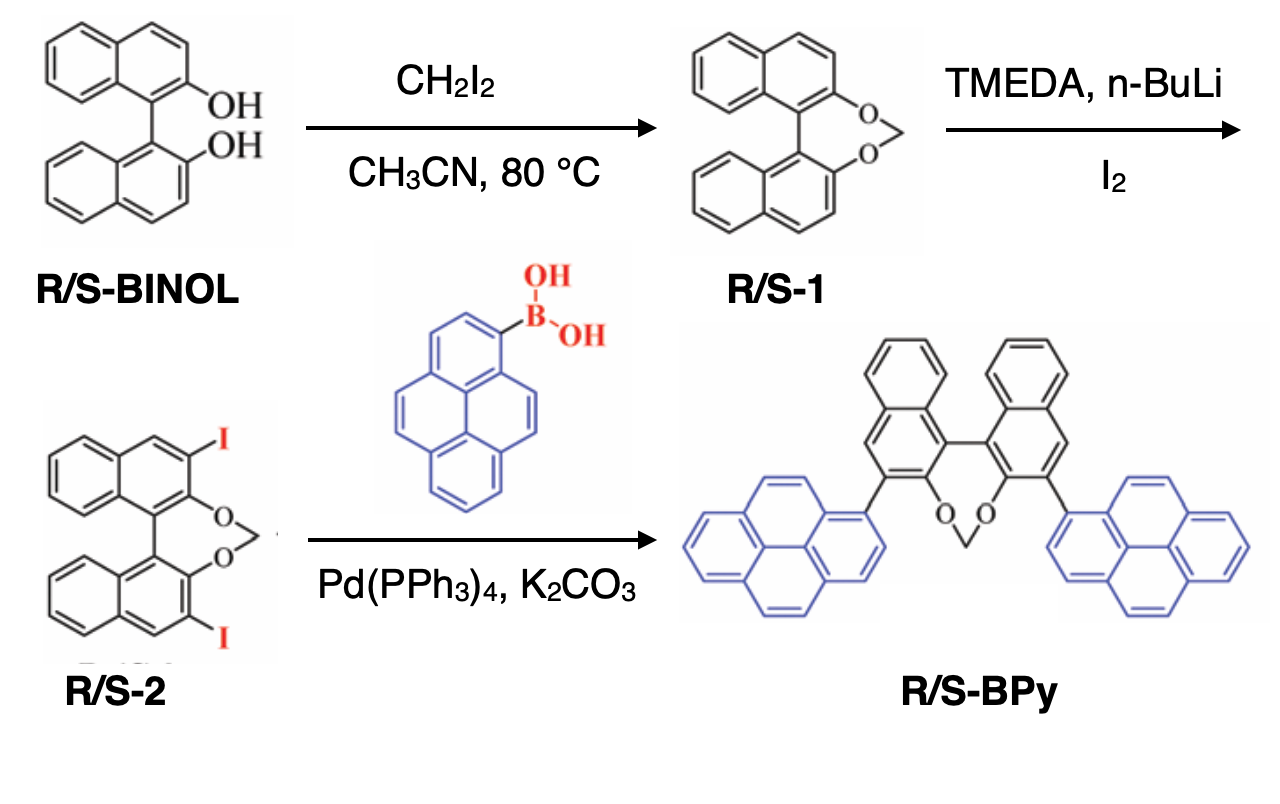


**(R)-[1,1'-binaphthalene]-[1,2,1,2-def]** (**R-1)**: Compound R-BINOL(2.86 g, 10 mmol), CH_2_I_2_(1 mL, 12 mmol), anhydrous K_2_CO_3_(4.14 g, 30 mmol), were dissolved in acetonitrile (60 mL). The reaction mixture was stirred at 80 °C for 8 h. Then, the mixture was cooled down to room temperature, subsequently extracted with CH_2_Cl_2_ (60 mL), washed with water (3 × 10 mL) and dried over Na_2_SO_4_. The precipitate was removed by filtration, and filtrate was evaporated under reduced pressure. The residue was purified by silica gel column chromatography (petroleum ether / ethyl acetate = 30/1, v/v) to afford **R-1** as a white solid (83% yield). ^1^H NMR (400 MHz, CDCl_3_) *δ* (ppm): 8.00 - 7.92 (m, 4H), 7.53 - 7.42 (m, 6H), 7.32-7.28 (m, 2H), 5.69 (s, 2H). **(R)-3,3'-Diiodo-[1,1'-binaphthalene]-[1,2,1,2-def]** (**R-2)**: Under an argon atmosphere, a 100 mL

Schlenk tube was charged with intermediate **R-1**(2 g, 6.7 mmol),*N*,*N*,*N*′,*N*′-

tetramethylethylenediamine (TMEDA) (4 mL, 26.81 mmol), and anhydrous diethyl ether (60 mL), the mixture was cooled to -78 °C, and the solution of *n*-BuLi (16 mL, 2.5 M in *n*-hexane) was added to the mixture dropwise for 30 minutes. After the mixture reacts at room temperature for 2 h, the mixture was cooled to -78 °C again, iodine (6.81 g, 26.81 mmol) was added to the mixture. Then, the reaction mixture was warmed to room temperature and stirred for overnight. 1 M sodium thiosulfate solution (30 mL) was added to the mixture. The organic layer was washed with water (3 × 10 mL) and dried over anhydrous Na_2_SO_4_. The precipitate was removed by filtration, and filtrate was evaporated under reduced pressure. The residue was purified by silica gel column chromatography (petroleum ether / ethyl acetate = 30/1, v/v) to afford **R-2** as a white solid (42% yield). ^1^H NMR (400 MHz, CDCl_3_) *δ* (ppm): 8.51 (s, 2H), 7.84 - 7.81 (d, *J* = 12 Hz, 2H), 7.48-7.41 (m, 4H), 7.32 - 7.28 (m, 2H), 5.68 (s, 2H).

**(R)-3,3'-di(1-pyrene)-[1,1'-binaphthalene]-[1,2,1,2-def][1,3]dioxepine (R-BPy)**: Under an argon atmosphere, a 50 mL Schlenk tube was charged with intermediate **R-2** (0.3 g, 0.55 mmol), 1-pyrenylboronic acid (0.41 g, 1.64 mmol), Pd(PPh_3_)_4_ (0.023 g, 0.02 mmol), anhydrous K_2_CO_3_ (0.23 g, 1.64 mmol), 1,4-dioxane (18 mL), and distilled water (2 mL). The reaction mixture was stirred at 90 °C for 24 h. Then, the mixture was cooled down to room temperature, subsequently extracted with CH_2_Cl_2_ (60 mL), washed with water (3 × 10 mL) and dried over Na_2_SO_4_. The precipitate was removed by filtration, and filtrate was evaporated under reduced pressure. The residue was purified by silica gel column chromatography (petroleum ether / ethyl acetate = 30/1, v/v) to afford **R-BPy** as a white solid (52% yield). ^1^H NMR (400 MHz, Chloroform-d) δ 8.24 -

7.77 (m, 24H), 7.63 - 7.44 (m, 4H), 4.82 (s, 2H).


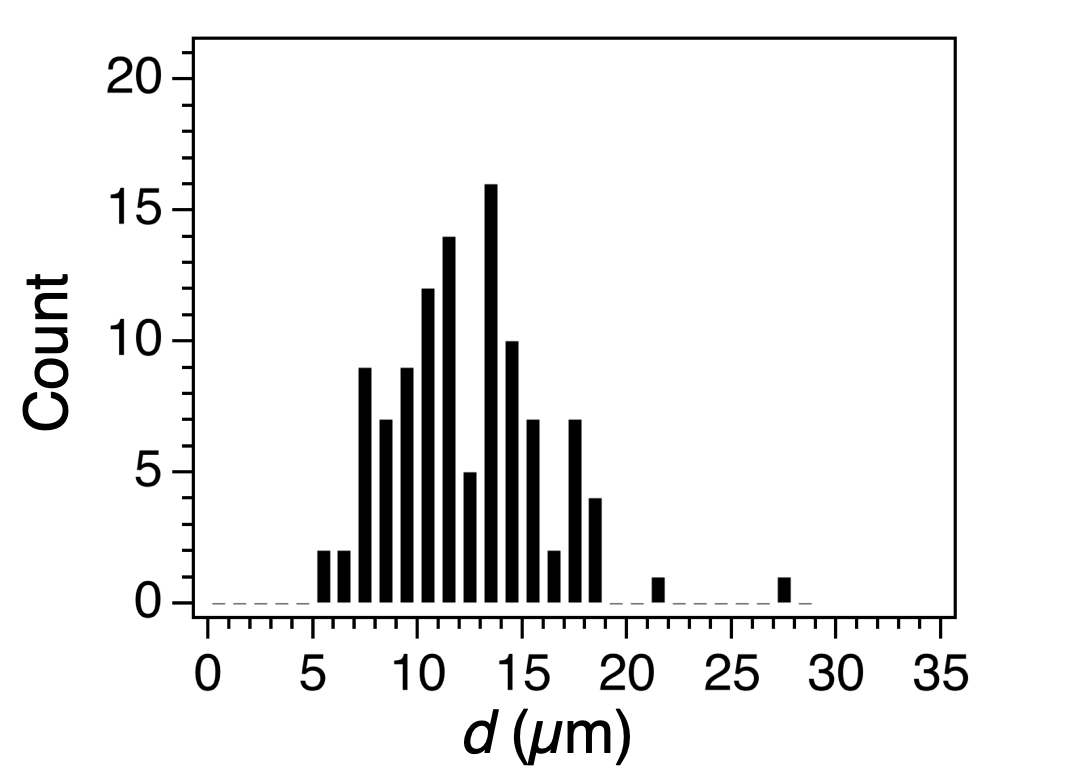


**Figure S1.** Histograms of *d* of each microsphere.


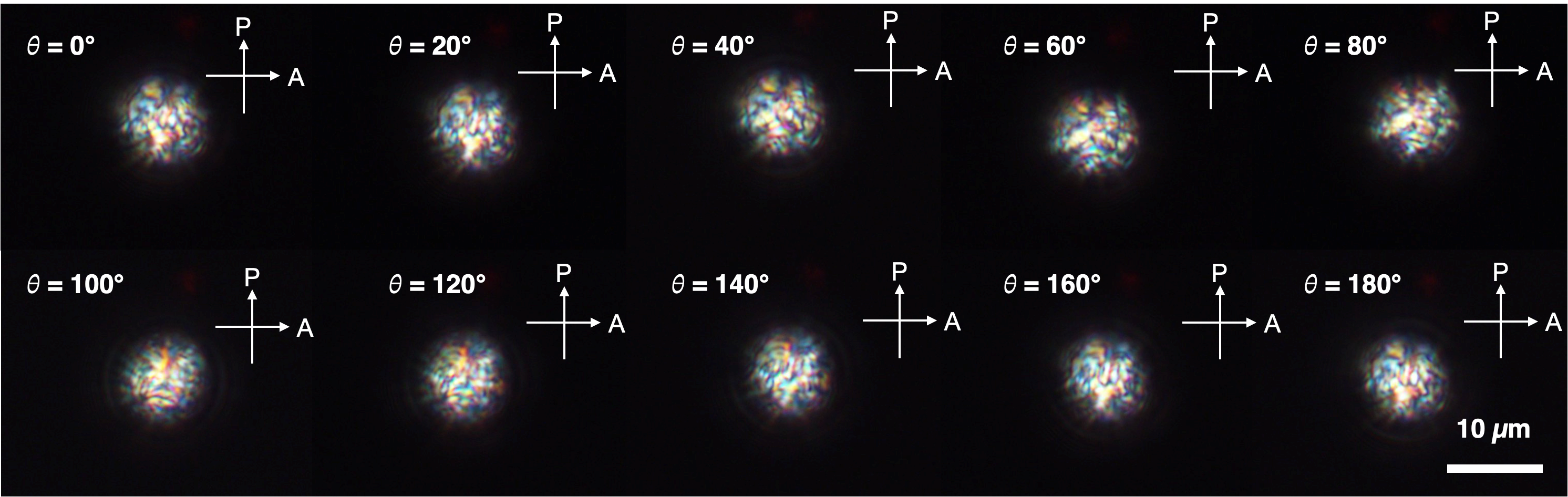


**Figure S2.** Angle-dependent POM textures of single **LCM^R-BPy^** operated with in-plane rotation.


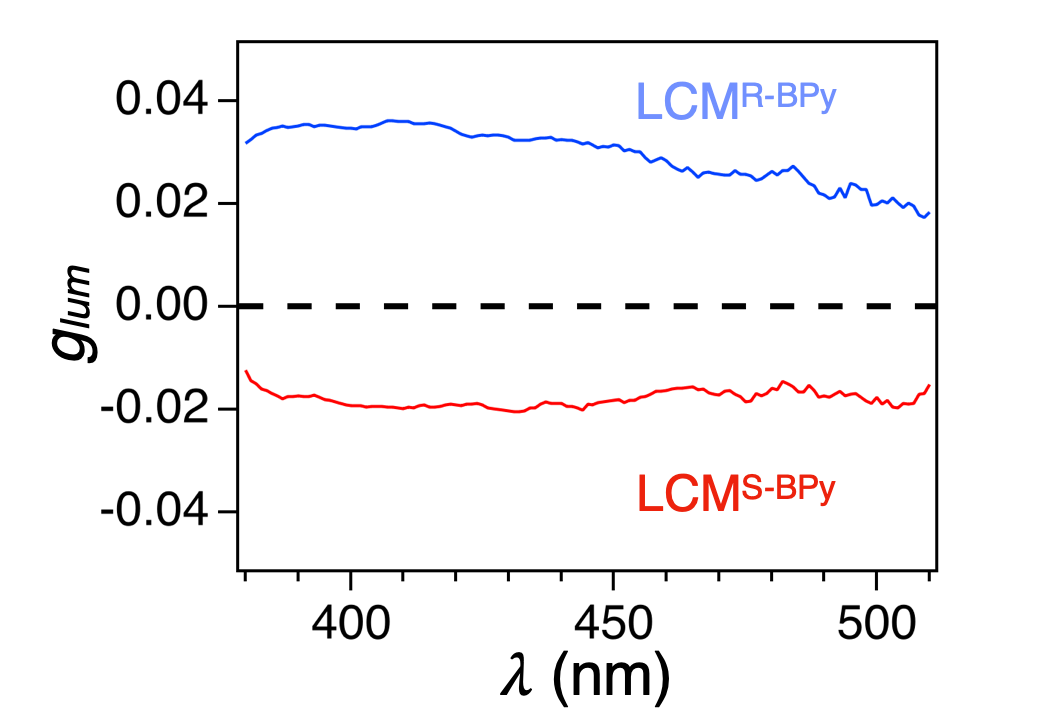


**Figure S3.** The *g*_lum_ value spectra (𝜆_ex_ = 350nm) of a glycerol suspension of **LCM^R-BPy^** (3wt% RBPy, blue curve) and **LCM^S-BPy^** (3wt% S-BPy, red curve).


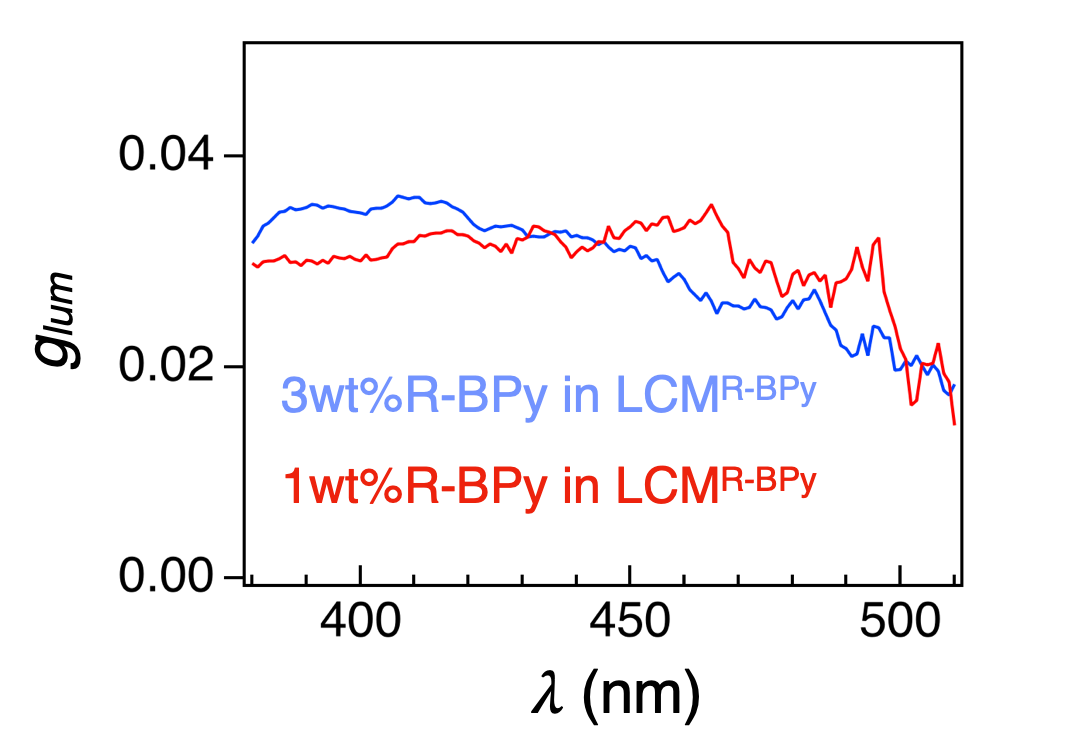


**Figure S4.** The *g*_lum_ value spectra (𝜆_ex_ = 350 nm) of a glycerol suspension of **LCM^R-BPy^** (3wt% RBPy, blue curve) and **LCM^R-BPy^** (1wt% R-BPy red curve).


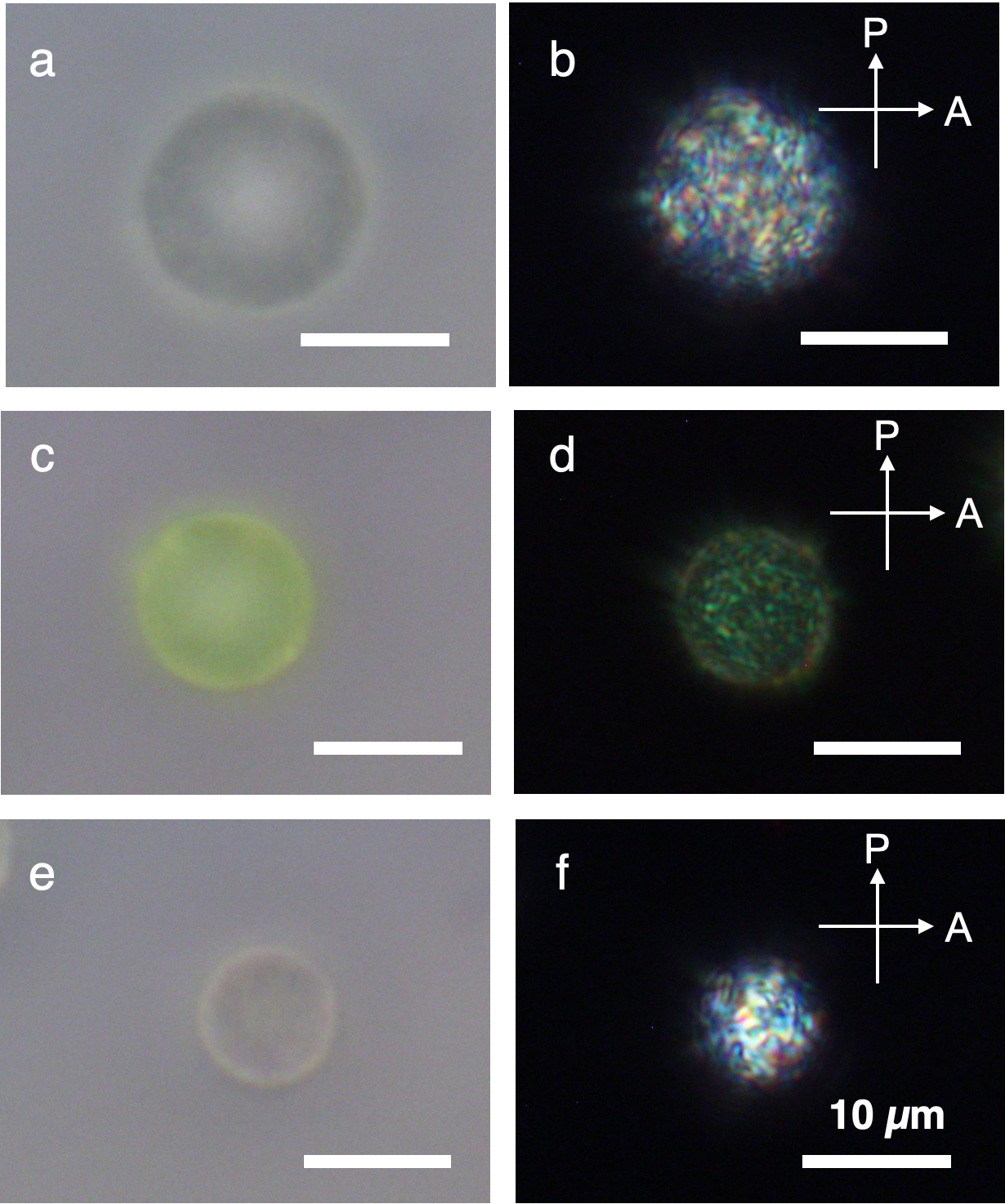


**Figure S5.** Optical micrographs and POM image of **LCM^Pe_R-BPy^** (a and b), **LCM^BPEA_R-BPy^** (c and d) and **LCM^H2OEP_R-BPy^** (e and f).


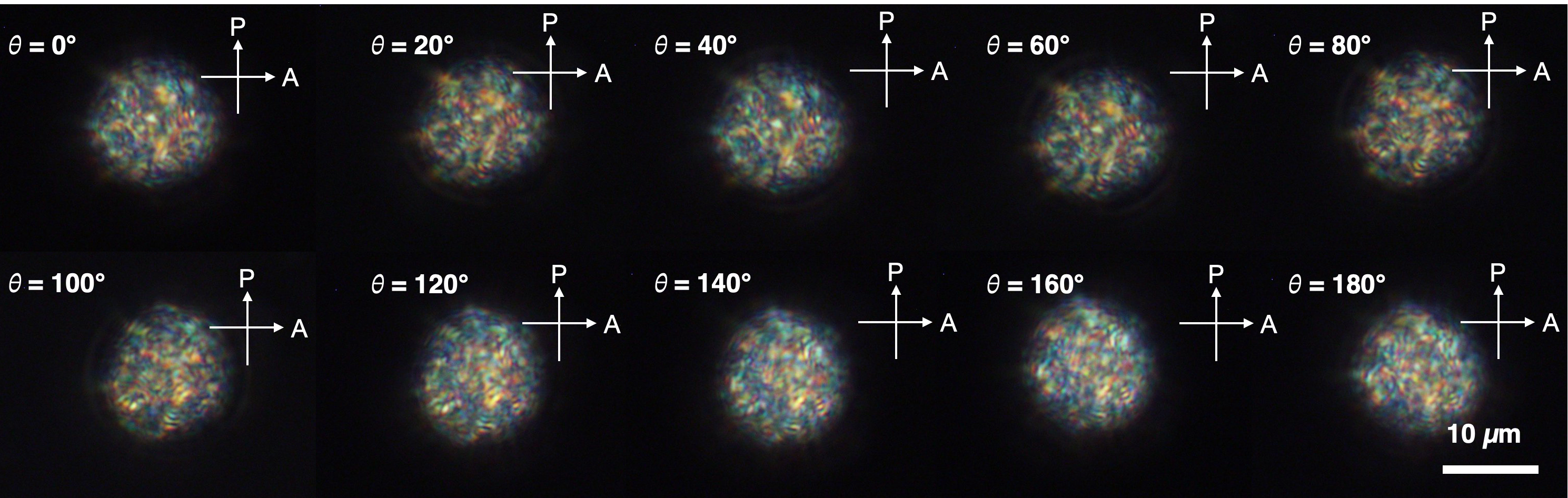


**Figure S6.** Micrographs of angle-dependent POM textures of a **LCM^Pe_R-BPy^** operated at in-plane rotation.


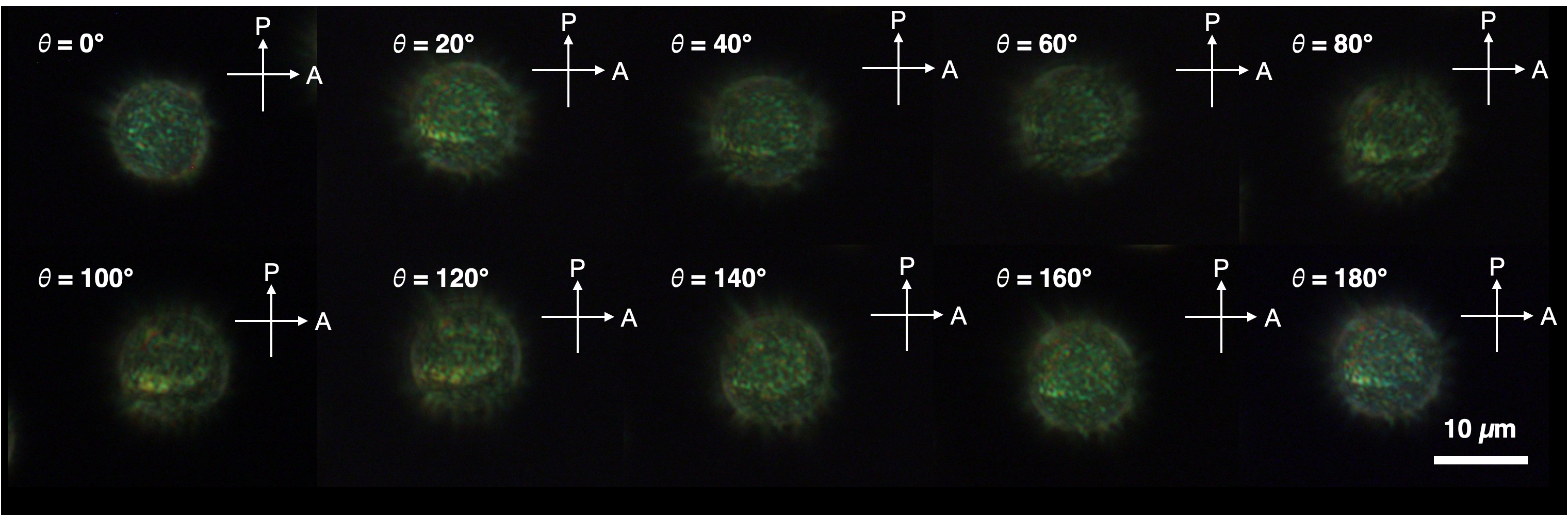
 **Figure S7.** Micrographs of angle-dependent POM textures of a **LCM^BPEA_R-BPy^** operated at inplane rotation.


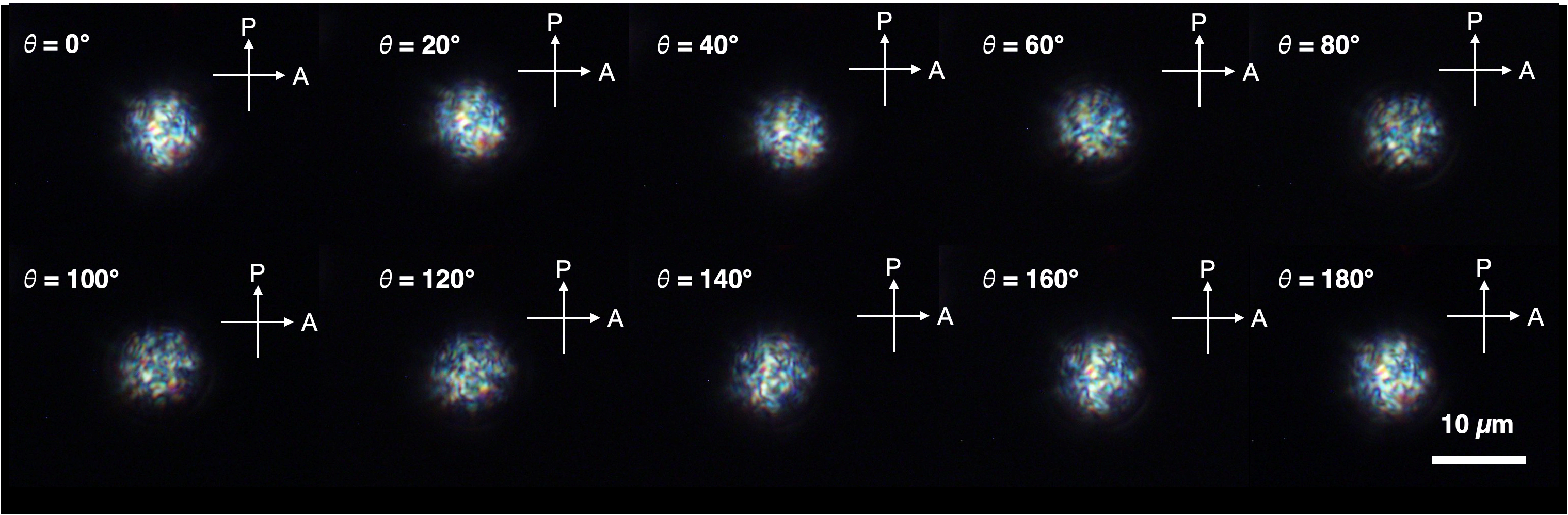


**Figure S8.** Micrographs of angle-dependent POM textures of a **LCM^H2OEP_R-BPy^** operated at inplane rotation.


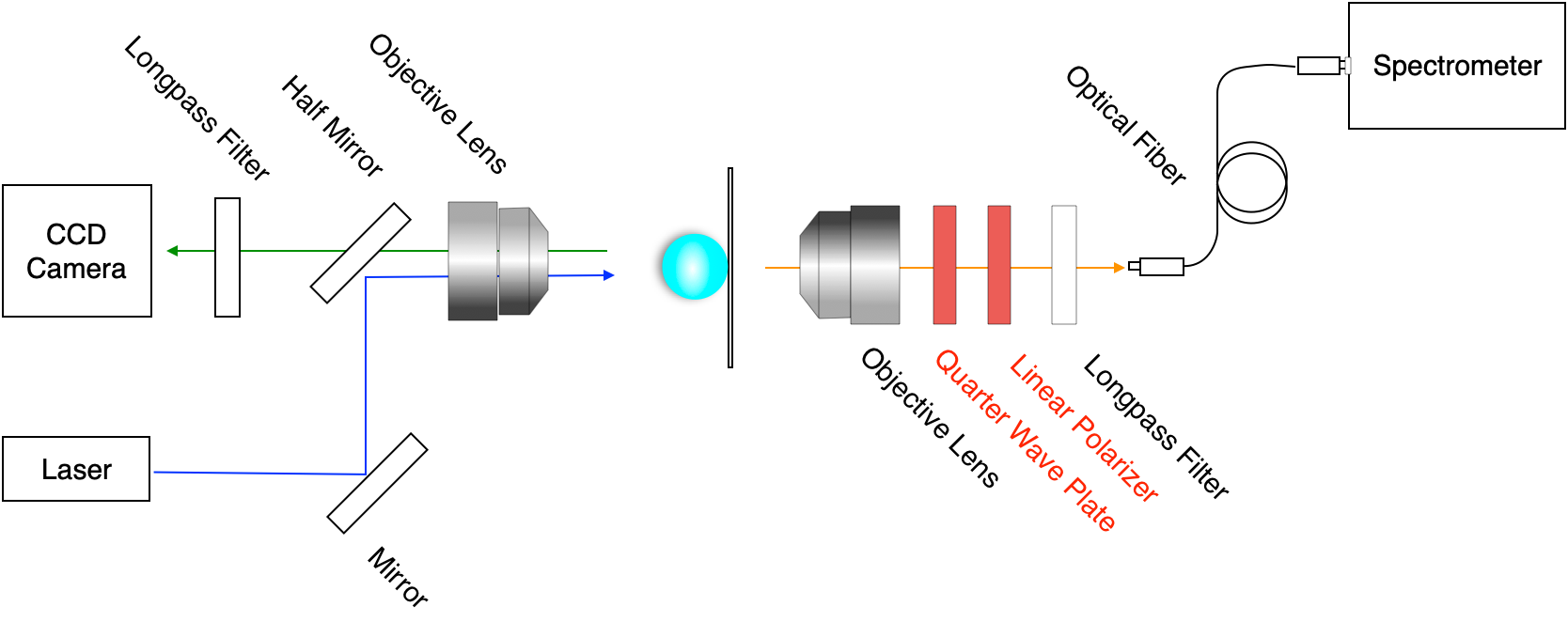


**Figure S9.** Schematic representations of the experimental setup of CPL measurement from a single microsphere. A single microsphere was excited by a depolarized CW laser (l_ex_ = 405 nm). PL from a single microsphere passes through a quarter wave plate (working range: 465−610 nm), a polarizer (working range: 400−700 nm), and a long-pass filter (working range: >450 nm) in a straight-line pass.


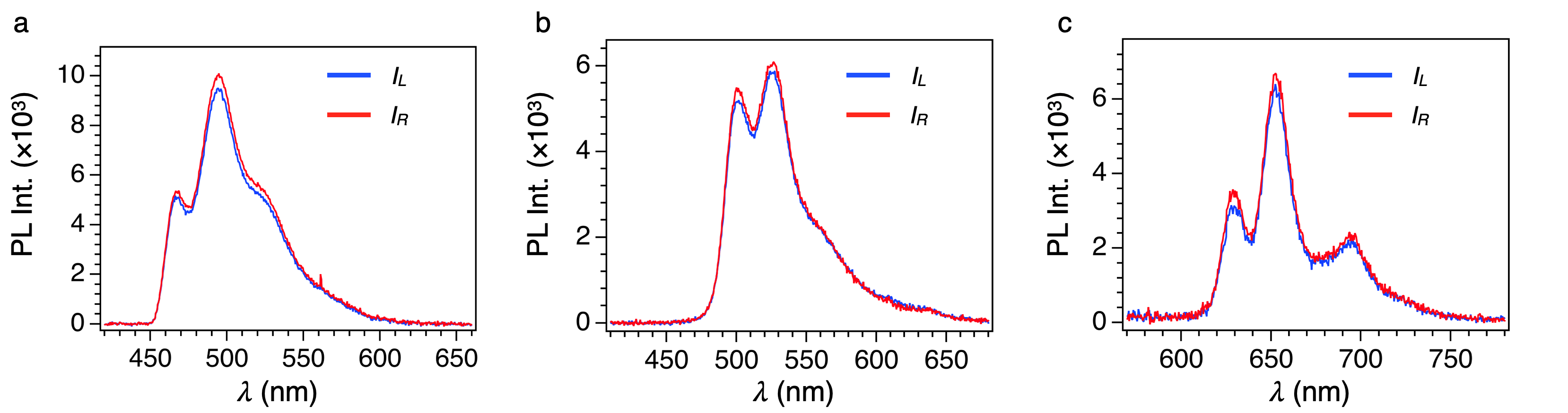


**Figure S10.** μ-CPL spectra of a single **LCM^Pe_S-BPy^** (a), **LCM^BPEA_S-BPy^** (b) and **LCM^H2OEP_S-BPy^** (c) with the direction of the polarizer at +45° (blue, *I_L_*) and −45° (red, *I_R_*). A single microsphere was excited by a depolarized CW laser (λ_ex_ = 405 nm).


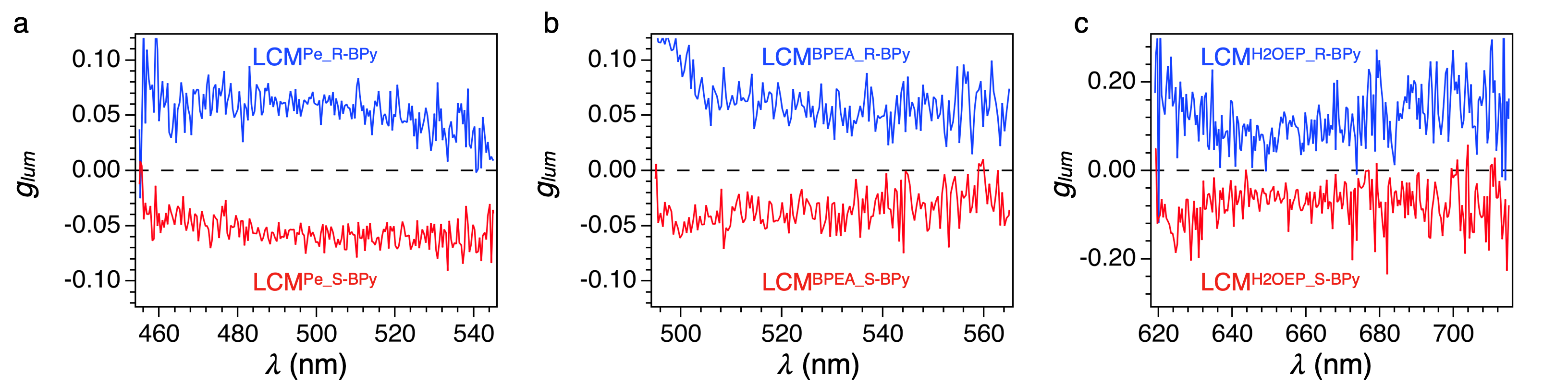


**Figure S11.** *glum* spectra of **LCMPe_R/S-BPy** (a), **LCMBPEA_R/S-BPy** (b) and **LCMH2OEP_R/S-BPy** (c)


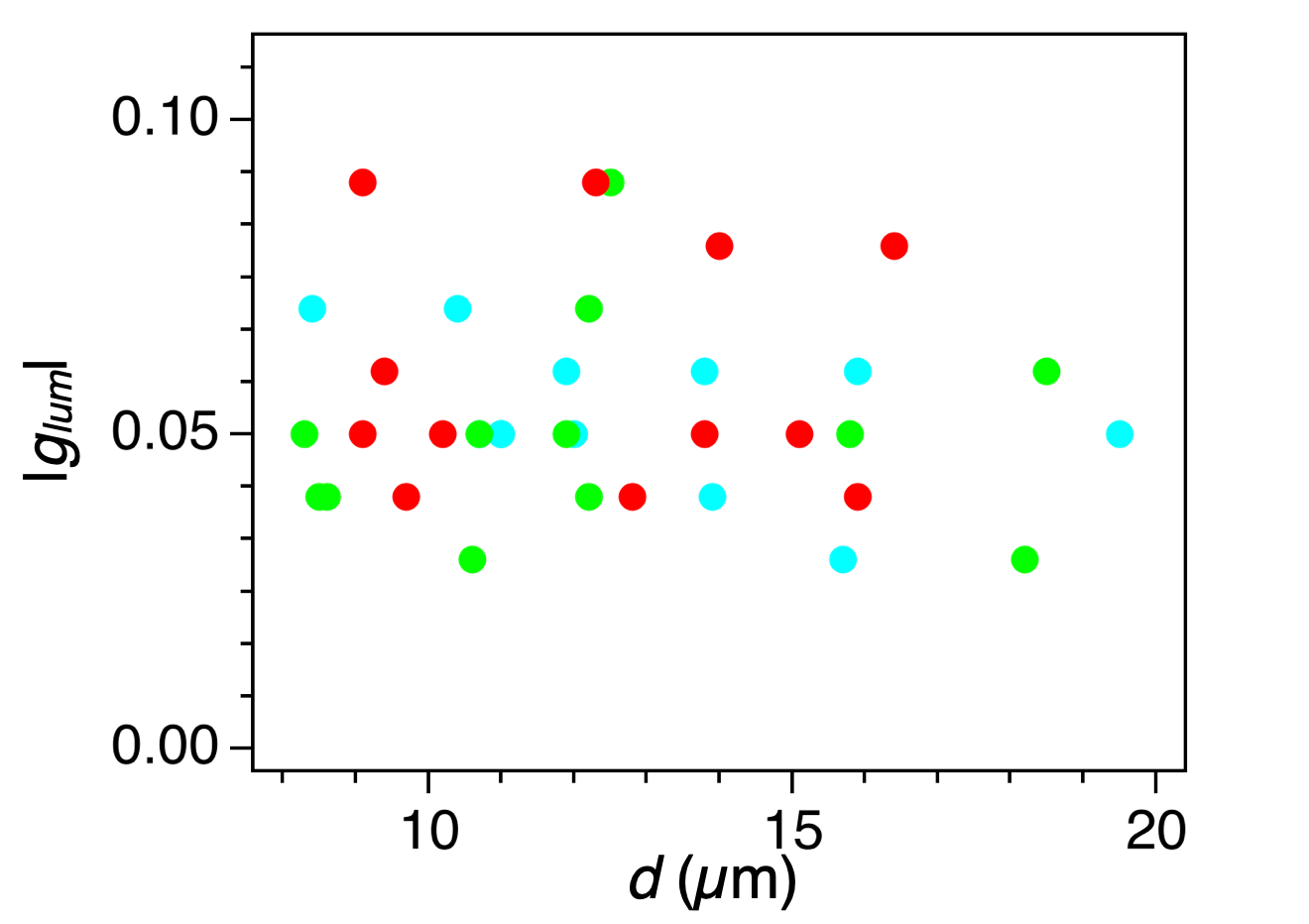


**Figure S12.** Scatter plot of diameter versus |*g*_lum_| value. Sky blue dot: **LCM^Pe_R/S-BPy^**, green dot:

**LCMBPEA_R/S-BPy**, red dot: **LCMH2OEP_R/S-BPy**.


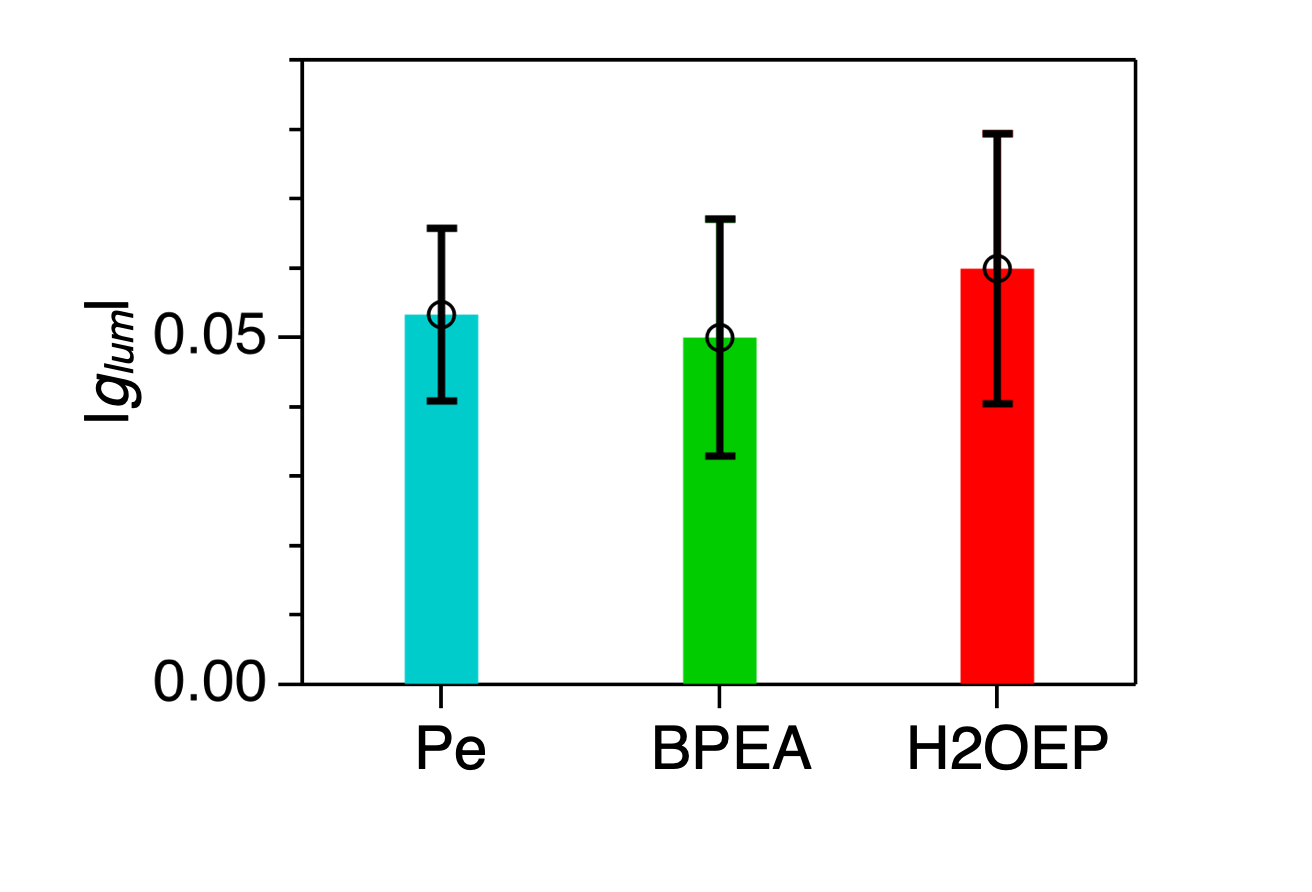


**Figure S13.** Histogram of the average |*g*_lum_| values for **LCM^Pe_BPy^**, **LCM^BPEA_BPy^**, and **LCM^H2OEP_BPy^**. Error bars represent standard deviation.


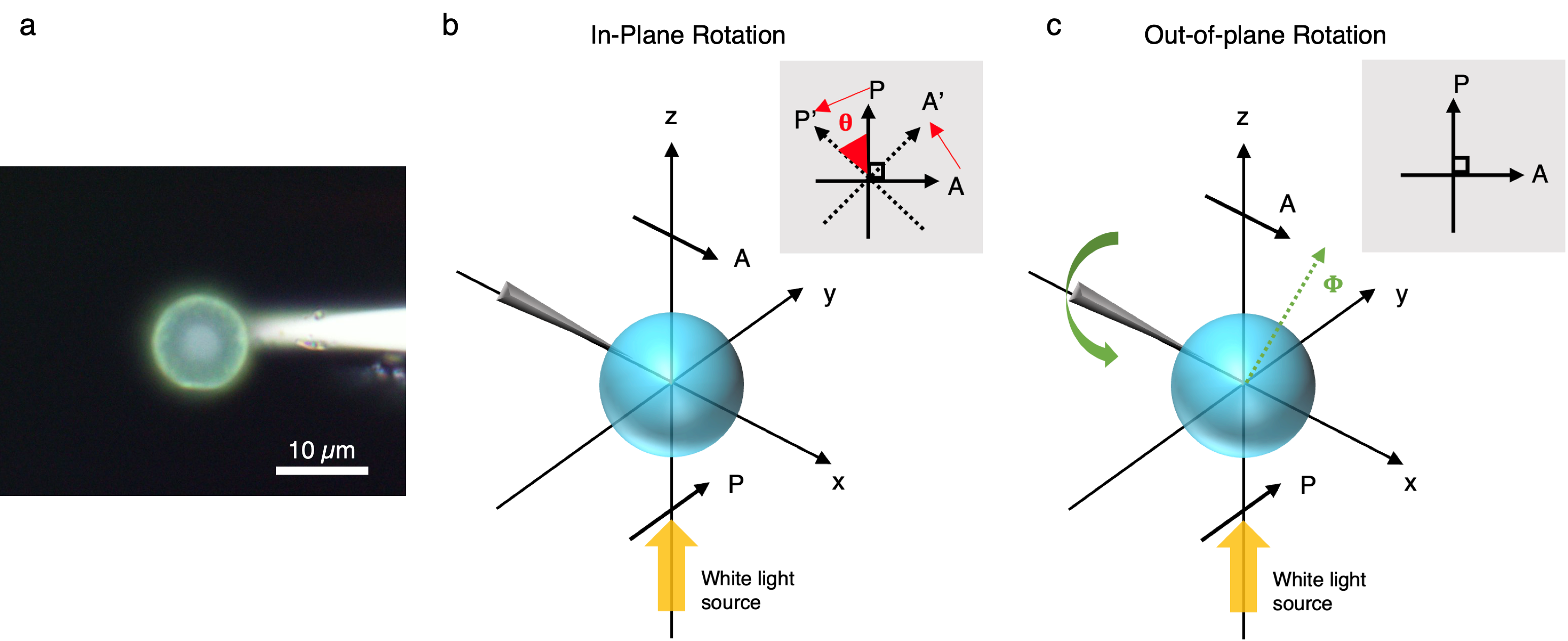


**Figure S14.** The optical microscope images of a single **LCM^Pe_R-BPy^** picked up by a sharp tungsten needle (a). Schematic representations of angle-dependent POM analysis of **LCM^Pe_R-BPy^** for (b) inplane (*θ*) rotation and (c) out-of-plane (𝛷) rotation. Inset image show the position of polarizer (P) and analyzer (A) from top view. For in-plane rotation, we keep the microsphere stationary and rotate P and Z at the same time. For out-of-plane rotation, we keep P and Z stationary and rotate needle to rotate the microsphere.


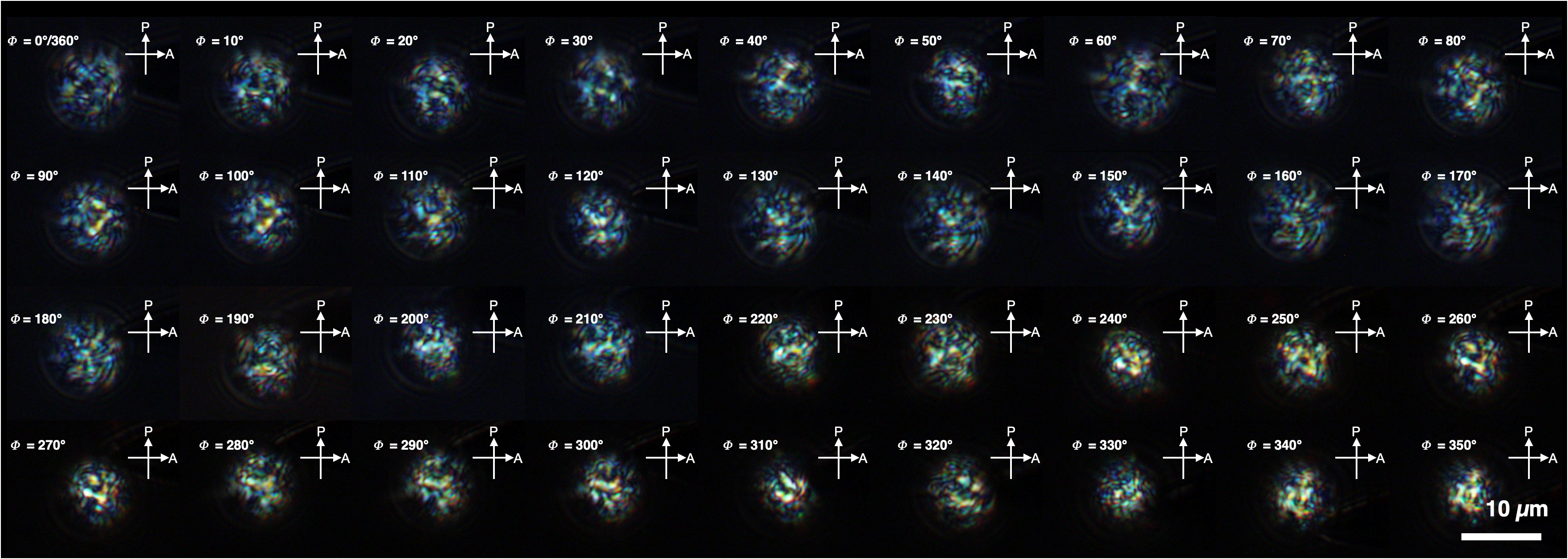


**Figure S15.** Micrographs of angle-dependent POM textures of a **LCM^Pe_R-BPy^** operated at out-ofplane rotation.


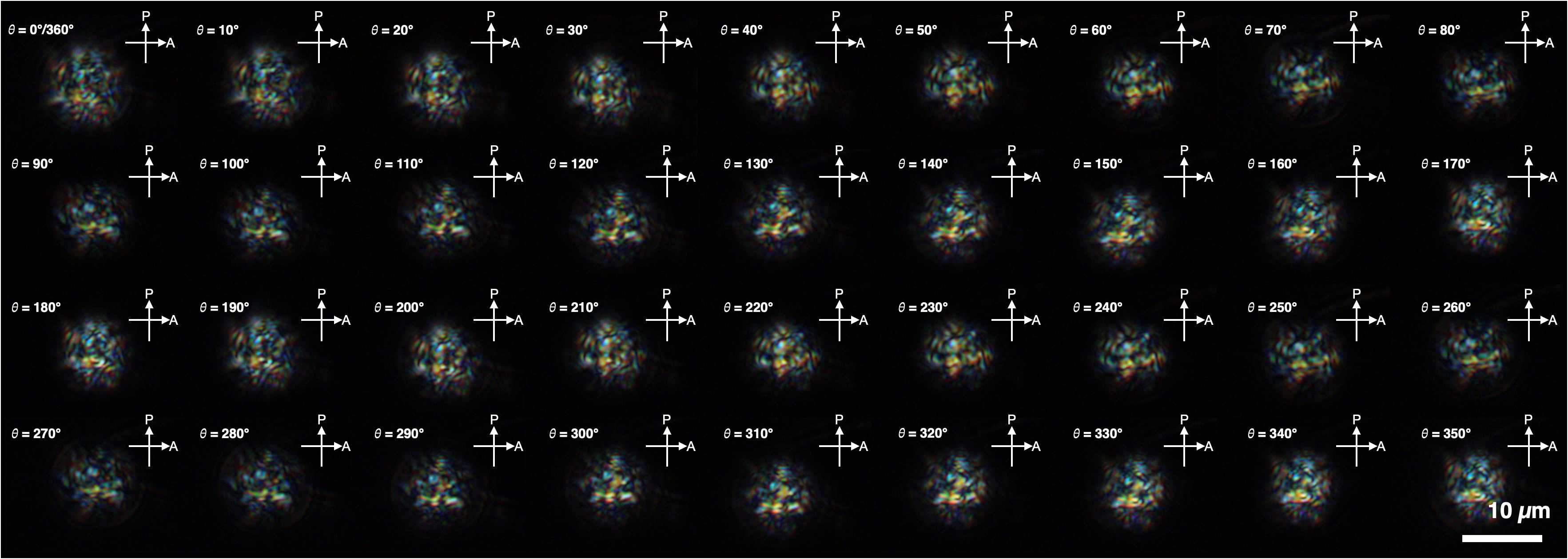


**Figure S16.** Micrographs of angle-dependent POM textures of a **LCM^Pe_R-BPy^** operated at in-plane rotation.


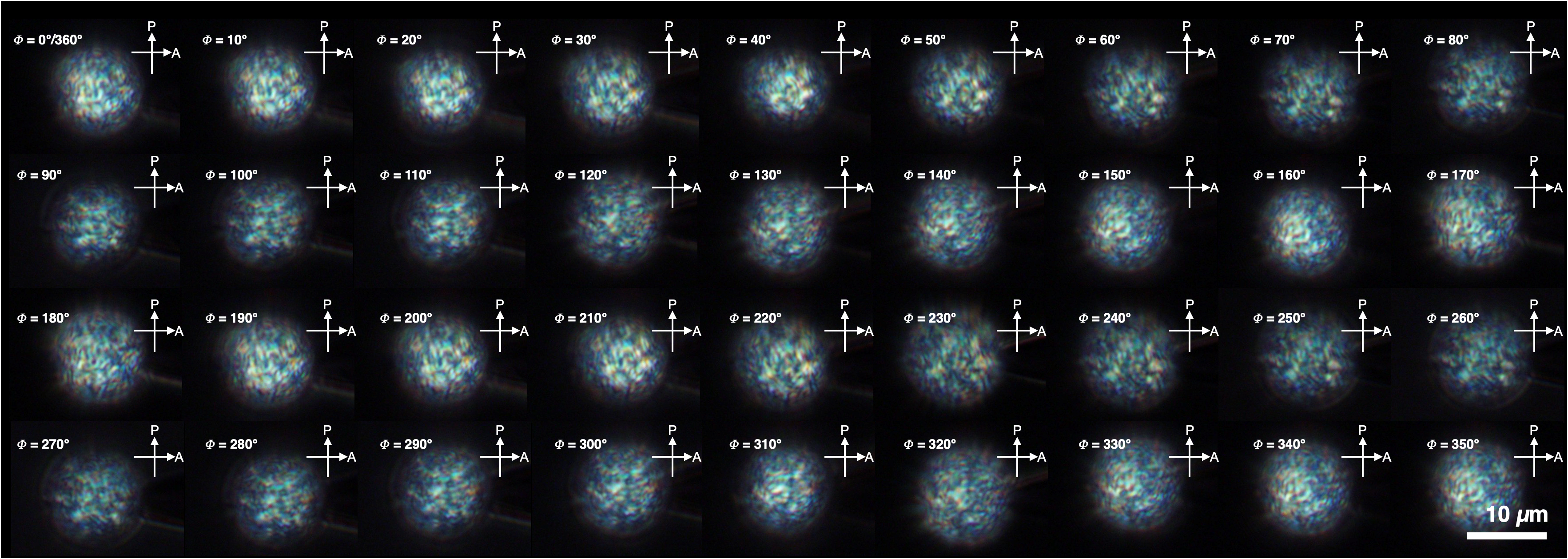


**Figure S17.** Micrographs of angle-dependent POM textures of a **LCM^Pe_S-BPy^** operated at out-ofplane rotation.


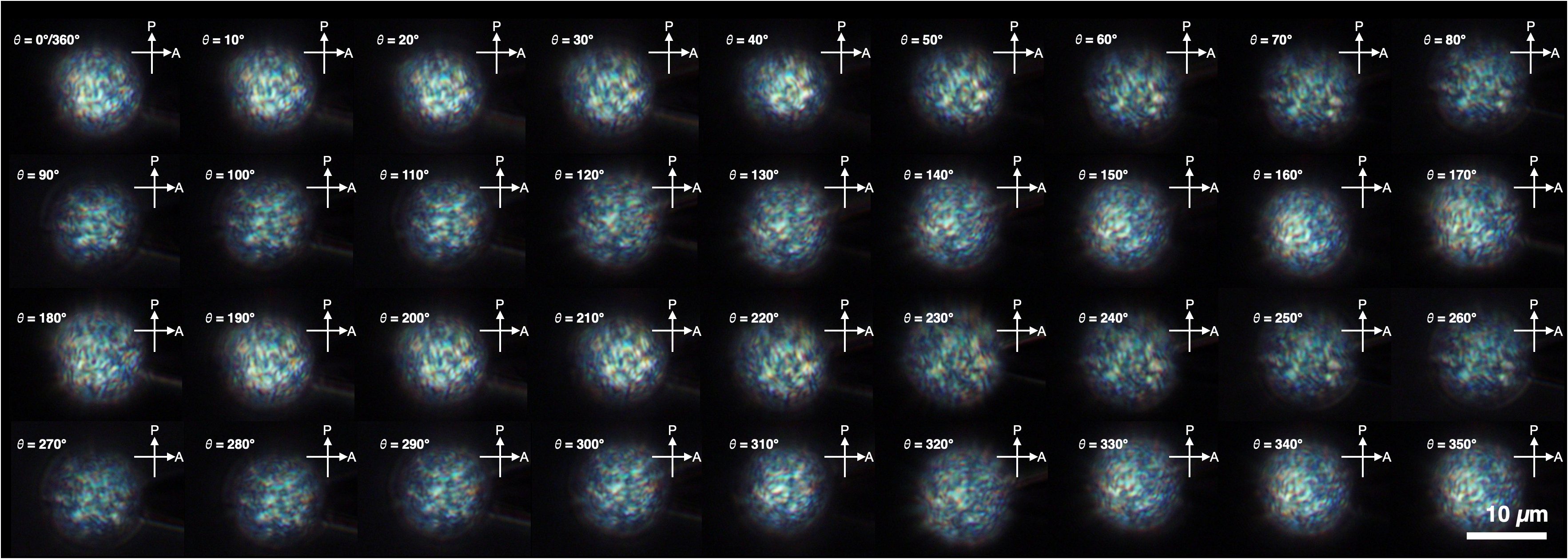


**Figure S18.** Micrographs of angle-dependent POM textures of a **LCM^Pe_S-BPy^** operated at in-plane rotation.


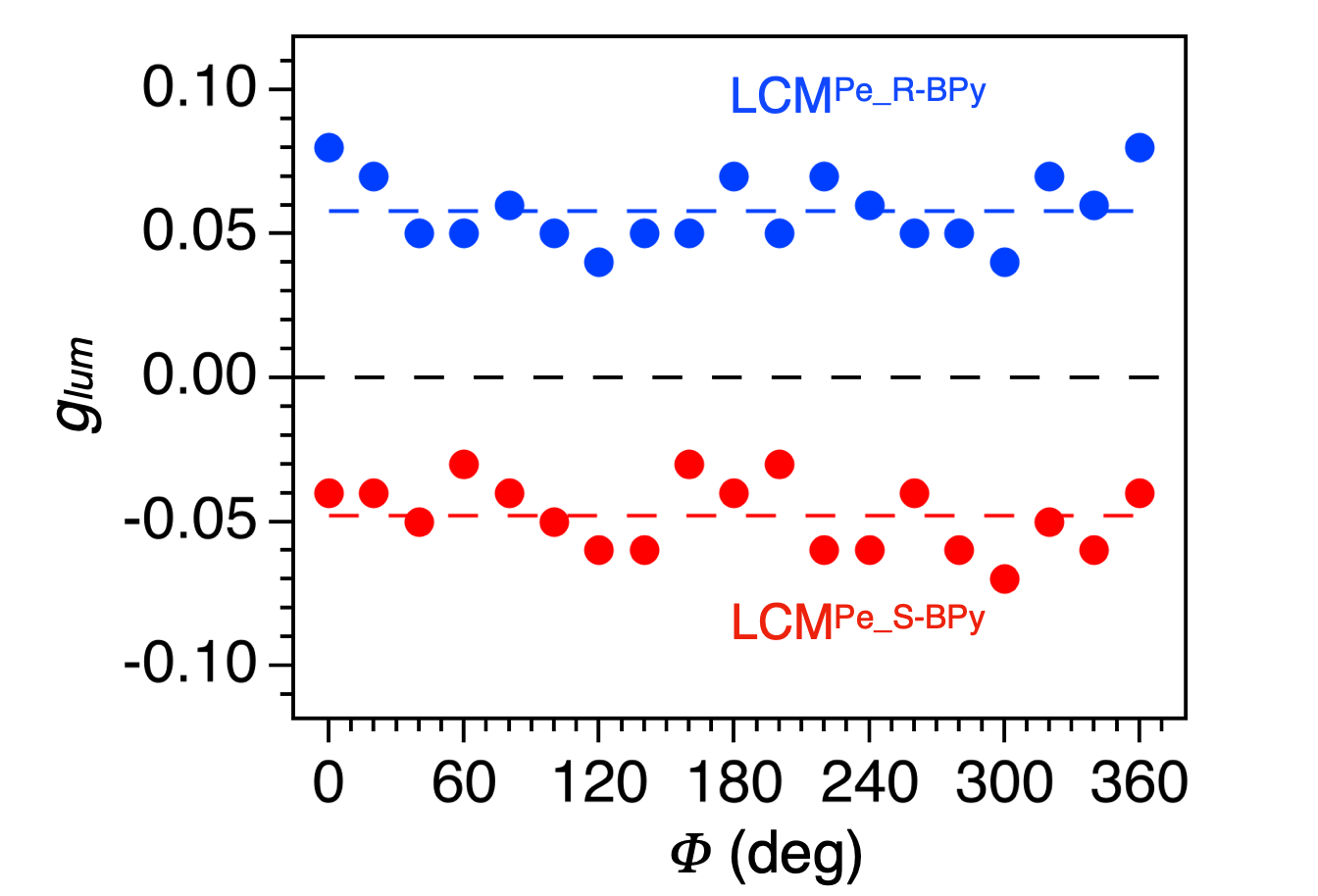


**Figure S19.** A plot of *g_lum_* value at 495 nm as a function of 𝛷.


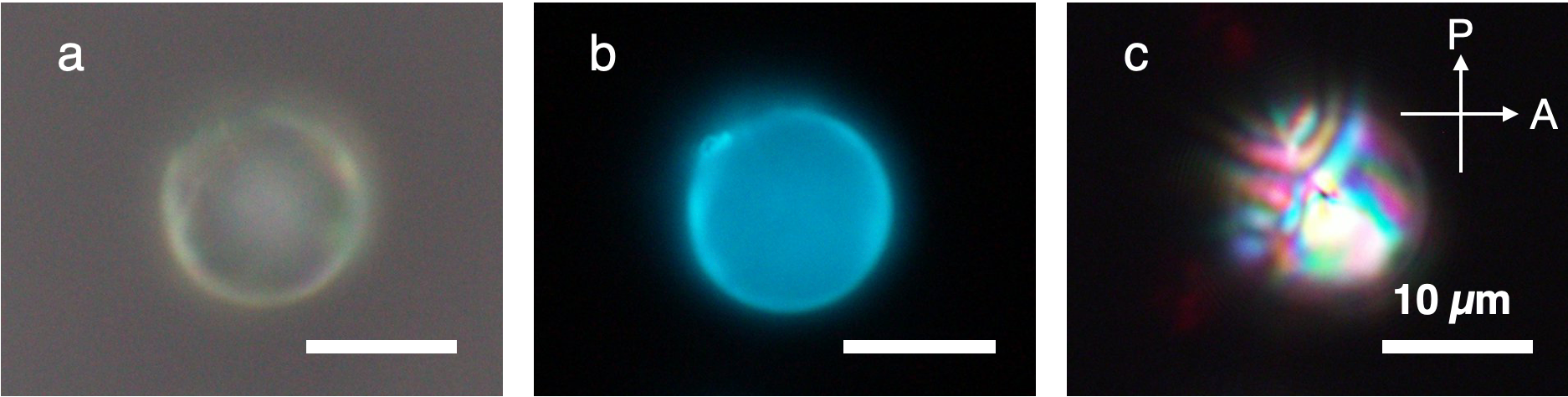
 **Figure S20.** Optical micrographs (a), fluorescence microscopy (b) and POM (c) images of **LCMPe_RM23**.


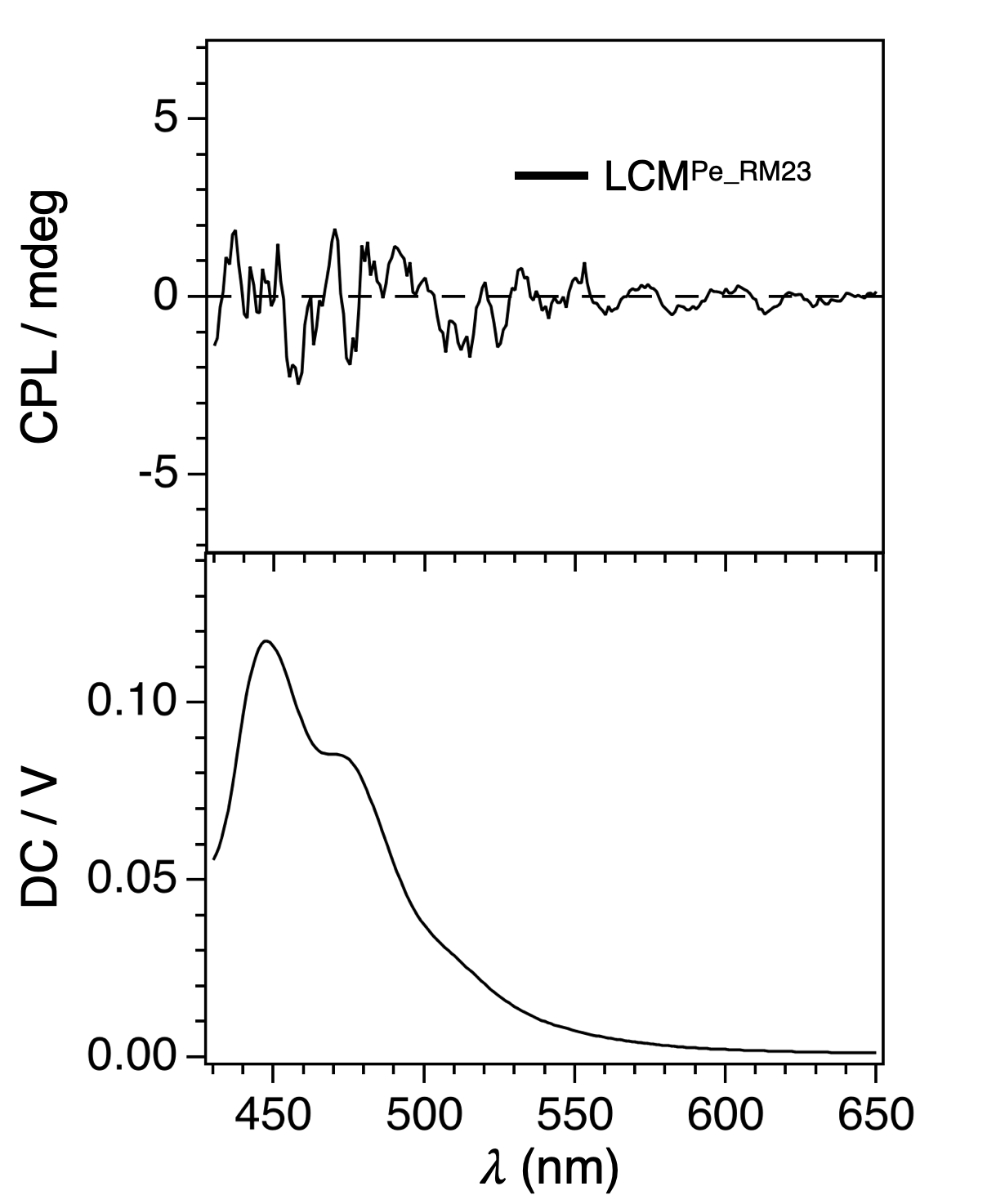


**Figure S21.** CPL spectrum (𝜆_ex_ = 390 nm) of glycerol suspension of **LCM^Pe_RM23^**


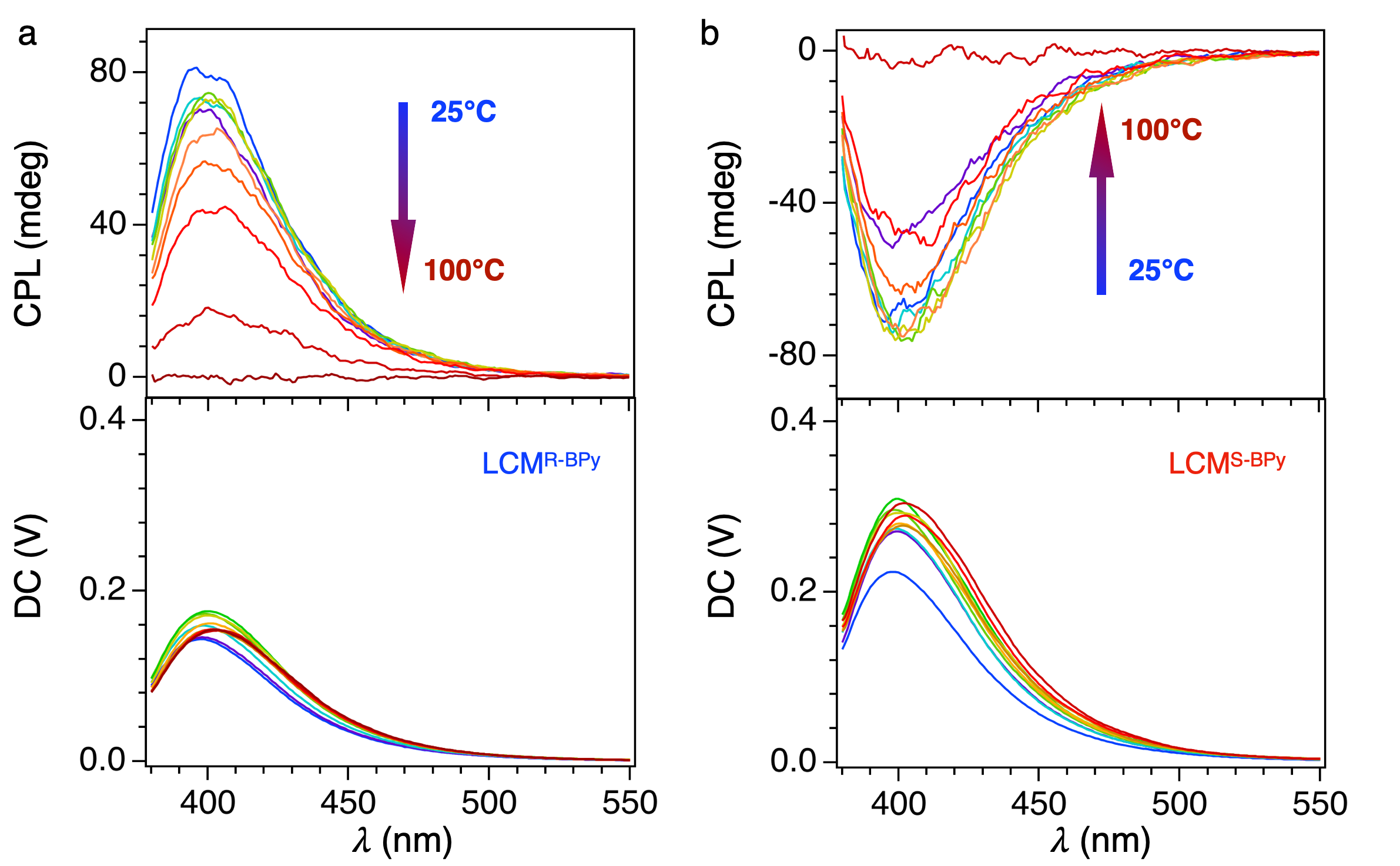


**Figure S22.** VT-CPL spectra (𝜆_ex_ = 350 nm) of **LCM^R-BPy^** (a) and **LCM^S-BPy^** (b).


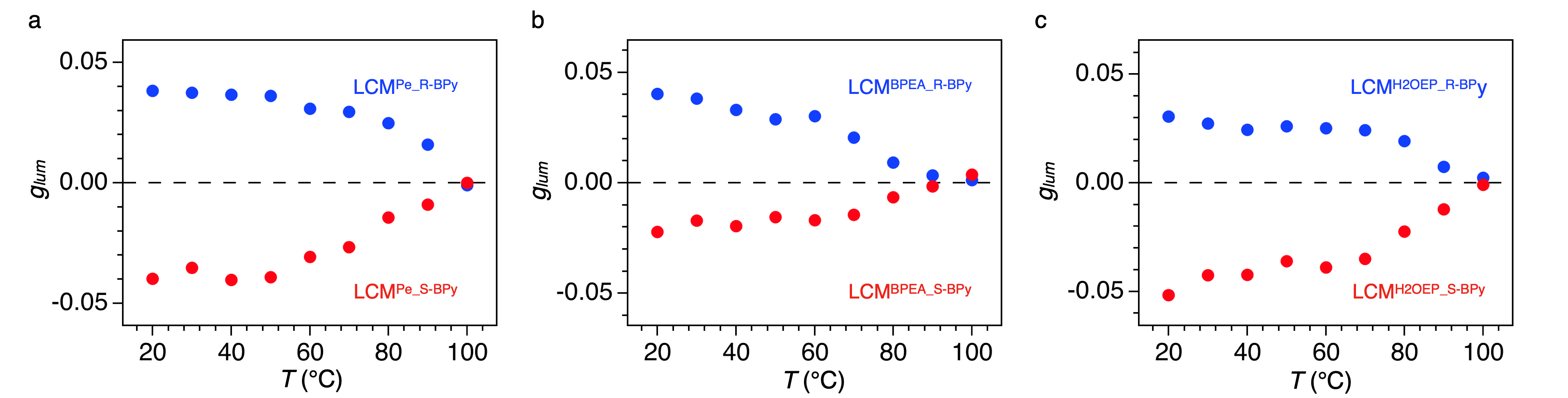


**Figure S23.** Temperature-dependent *g_lum_* for **LCM^Pe_R-BPy^** (a, blue, 𝜆_ex_ = 390 nm), **LCM^Pe_S-BPy^** (a, red, 𝜆_ex_ = 390 nm), **LCM^BPEA_R-BPy^** (b, blue, 𝜆_ex_ = 425 nm), **LCM^BPEA_S-BPy^** (b, red, 𝜆_ex_ = 425 nm), and **LCM^H2OEP_R-BPy^** (c, blue, 𝜆_ex_ = 405 nm), **LCM^H2OEP_S-BPy^** (c, red, 𝜆_ex_ = 405 nm).


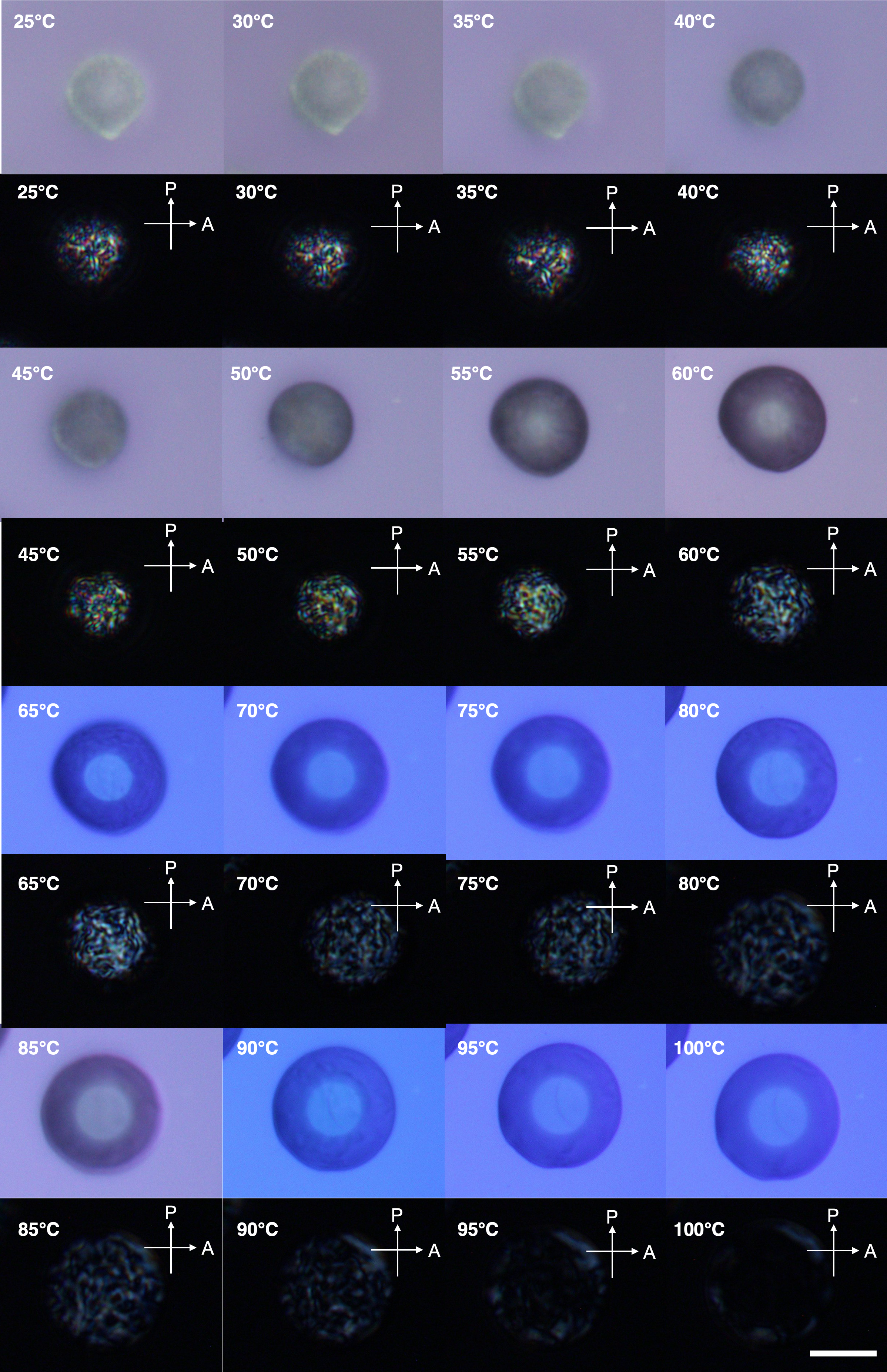


**Figure S24.** Variable-temperature optical micrographs and POM images of **LCM^R-BPy^**.


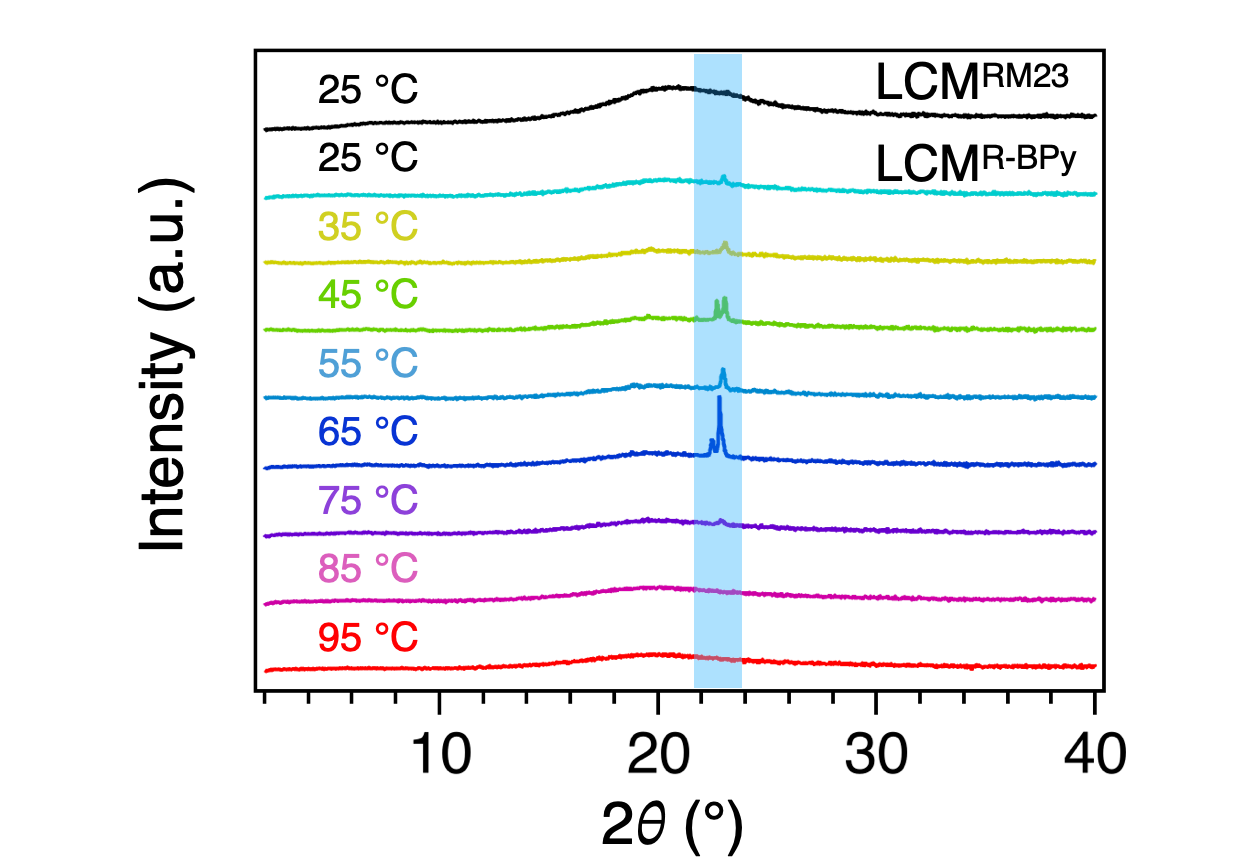


**Figure S25.** XRD of **LCM^RM23^** and variable-temperature XRD spectra of **LCM^R-BPy^**.

**Table S1.** Summary of *g*_lum_ from Various Microspheres


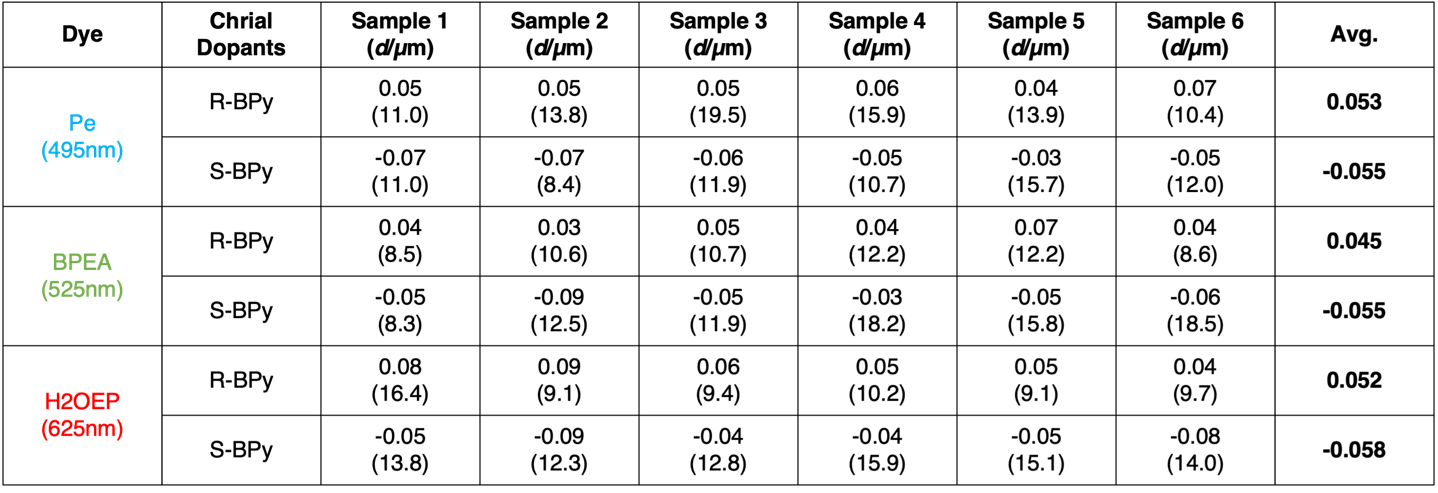


# Supporting Reference

[S1]. X. Y. Zhang, Z. R. Xu, Y. Zhang, Y. W. Quan, Y. X. Cheng, *J. Mater. Chem. C,* **2020**, 8, 15669-15676.
